# Segmented projection image translation for large-area, high-resolution vat photopolymerization

**DOI:** 10.1126/sciadv.aef7779

**Published:** 2026-11-11

**Authors:** Jize Dai, Jason M. Kronenfeld, Dan Ilyn, Christopher Linhardt, Philip R. Onffroy, Ian A. Coates, Colin Crown, Michelle Chen, Jacob Dobson, Max A. Saccone, John R. Tumbleston, Maria T. Dulay, Joseph M. DeSimone

**Affiliations:** ^1^ Department of Mechanical Engineering, Stanford University., 318 Campus Drive, Stanford, CA 94305-5427, USA.; ^2^ Department of Chemistry, Stanford University, 318 Campus Drive, Stanford, CA 94305-5427, USA.; ^3^ Department of Radiology, Stanford University, 318 Campus Drive, Stanford, CA 94305-5427, USA.; ^4^ Department of Chemical Engineering, Stanford University, 318 Campus Drive, Stanford, CA 94305-5427, USA.; ^5^ Department of Mechanical Engineering, University of Colorado Boulder, 1111 Engineering Drive 427 UCB, Boulder, CO 80309-0552, USA.

## Abstract

High-resolution projection-based vat photopolymerization (VP) 3D printing enables rapid fabrication of highly detailed polymeric structures. However, expanding the build area traditionally compromises resolution, creating a fundamental trade-off between projected pixel size and printable area due to fixed digital micromirror device (DMD) pixel counts. Herein, we introduce segmented projection image translation (SPIN), a simple implementation strategy that expands the effective projection area by applying controlled orbital translation to an off-axis objective lens within the optical infinity space. In this work, we demonstrate a projection-based VP printer implemented with SPIN that expands the effective build area by 4.8-fold, from a native field of 60.4 square millimeters to an expanded domain of 290.5 square millimeters. The SPIN-enabled printer produces isolated line features as thin as 9.8 ± 2.2 micrometers and fabricates intricate three-dimensional truss structures with features as small as 30 micrometers. These results validate a practical route for extending microscale projection-based VP printing to larger build areas while preserving feature fidelity across stitched exposure fields.

## INTRODUCTION

Additive manufacturing (AM) has redefined how engineering devices are fabricated by enabling direct access to geometric design spaces that are conventionally difficult or impossible to realize using prevailing manufacturing approaches such as molding, machining, and lithography. AM enables the fabrication of complex, three-dimensional (3D) architectures with precisely defined internal features, spatial gradients, and interconnected negative space. Such geometric freedom enables function to be digitally parameterized and directly realized via printed architecture. As a result, AM has enabled a broad class of systems ranging from architected materials (*1*–*3*) and microrobotic components (*3*–*6*) to biomedical devices (*7*–*10*) and energy storage systems (*11*, *12*), whose operation depends on controlled 3D transport, surface-area density, and structural hierarchy.

Among existing AM modalities, projection-based vat photopolymerization (VP) approaches, notably digital light processing (DLP) (*13*, *14*) and continuous liquid interface production (CLIP) (*15*), enable the rapid fabrication of high-resolution 3D polymeric structures by exposing entire 2D layers simultaneously. Despite continual advancements in throughput speed, the maximum printable area and native pixel size in these systems are constrained by the fixed total pixel count of the light engine’s digital micromirror device (DMD) defining the projected image. This invariant pixel count dictates that any optical modification to enhance spatial resolution (e.g., reducing the effective pixel size) inherently results in a contraction of the projected build area (*16*). This fundamental trade-off forces designers to choose between high resolution and large-area printing.

Current approaches to expanding the build area of projection-based printers commonly rely on mechanical translation of either the light source assembly (*17*–*19*) or the build platform (*20*, *21*) to expose adjacent regions within a layer (see section S1 for visual comparisons of different methods). Although this strategy can preserve the native projected pixel size, it introduces several practical limitations. Translating a heavy light engine or projection assembly introduces stitching errors and in-plane registration mismatches across adjacent exposure fields; mitigating these defects requires highly precise mechanical motion, complex software compensation, or both, driving up overall system cost (*17*–*19*). Alternatively, translating the build platform directly disturbs the resin vat, mandating extended dwell times for fluid quiescence before the next exposure segment can be projected, which inherently accumulates into extended fabrication times (*20*). Advanced optical scaling alternatives, such as multiprojector arrays or scanning-mirror architectures, attempt to expand the build area while avoiding these mechanical translation drawbacks (*22*–*25*) but mandate intensive interprojector and optical calibration profiles as well as expensive or one-off auxiliary hardware that limit low-cost system replication. Furthermore, in high-resolution regimes where the individual projected image footprint is smaller than the physical lens diameter, multiprojector or multi–optical path configurations are geometrically barred from overlapping adjacent fields due to spatial mechanical interference, restricting these setups to simple pattern duplication rather than true continuous expansion of a singular build field (*25*). Together, these fundamental limitations motivate an alternative optomechanical approach capable of expanding the effective build area while avoiding inertia-intensive translations, preserving micrometer-scale resolution, reducing flow-induced delays, and maintaining practical system replicability.

Herein, we report a simple, rapidly implementable technique, called segmented projection image translation (SPIN), that expands the effective build area of projection-based VP printers without translating the light engine or build platform. By pairing conventional microscope optics with tilt-shift lens principles, SPIN introduces a controlled orbital translation of an off-axis objective lens within the optical infinity space. This lateral configuration displaces the projected image in a controlled, repeatable, segmented manner across the build plane without altering the position of the light engine or disturbing fluid resin equilibrium. By limiting motion to the small objective lens through a compact rotary mechanism, SPIN reduces inertia and lowers system cost by avoiding large, high-precision linear stages or gantries. In this work, we demonstrate a 4.8-fold expansion of the effective build area, scaling a singular exposure projection field from 60.4 to 290.5 mm^2^ while preserving a calculated native pixel size of 3.86 μm. This expanded build area retains microscale printing capability, enabling isolated line features as thin as 9.8 μm and intricate 3D truss structures with features as small as 30 μm. Tensile testing further showed no statistically significant differences in ultimate tensile strength or Young’s modulus across stitched segment boundaries, indicating mechanical uniformity across these regions. This methodology provides an accessible, cost-effective paradigm for the scalable manufacturing of next-generation microelectronics cooling systems, high-surface-area battery architectures, and biosynthetic organ vascularization networks.

## RESULTS

### Implementation and mechanical design of SPIN

In the printer architecture, the SPIN mechanism is installed between the light engine and the resin vat (see section S2 to S4 for the experimental setup, structural details of SPIN, and bill of materials, respectively). SPIN expands the build area through a simple orbital translation of the objective. Before the installation, the image projected via the objective is aligned with the primary optical axis, as shown in Fig. 1Aa. After installation, the objective is radially displaced from the primary optical axis by a distance, d, resulting in a corresponding lateral shift of the projected image by the same distance while preserving its spatial resolution (Fig. 1Ab). The light engine lens and objective used in this work had exit and entrance pupil diameters of 22 and 12 mm, respectively. This allowed the objective to be shifted by ∼5 mm (experimentally measured as an average of 4.8 mm) while maintaining sufficient clear aperture to fully illuminate the build area. During fabrication, the SPIN is programmatically controlled to orbit the offset objective and thus the projected image, about the optical axis, resulting in a notably larger projectable region (Fig. 1Ac).

**Fig. 1.**
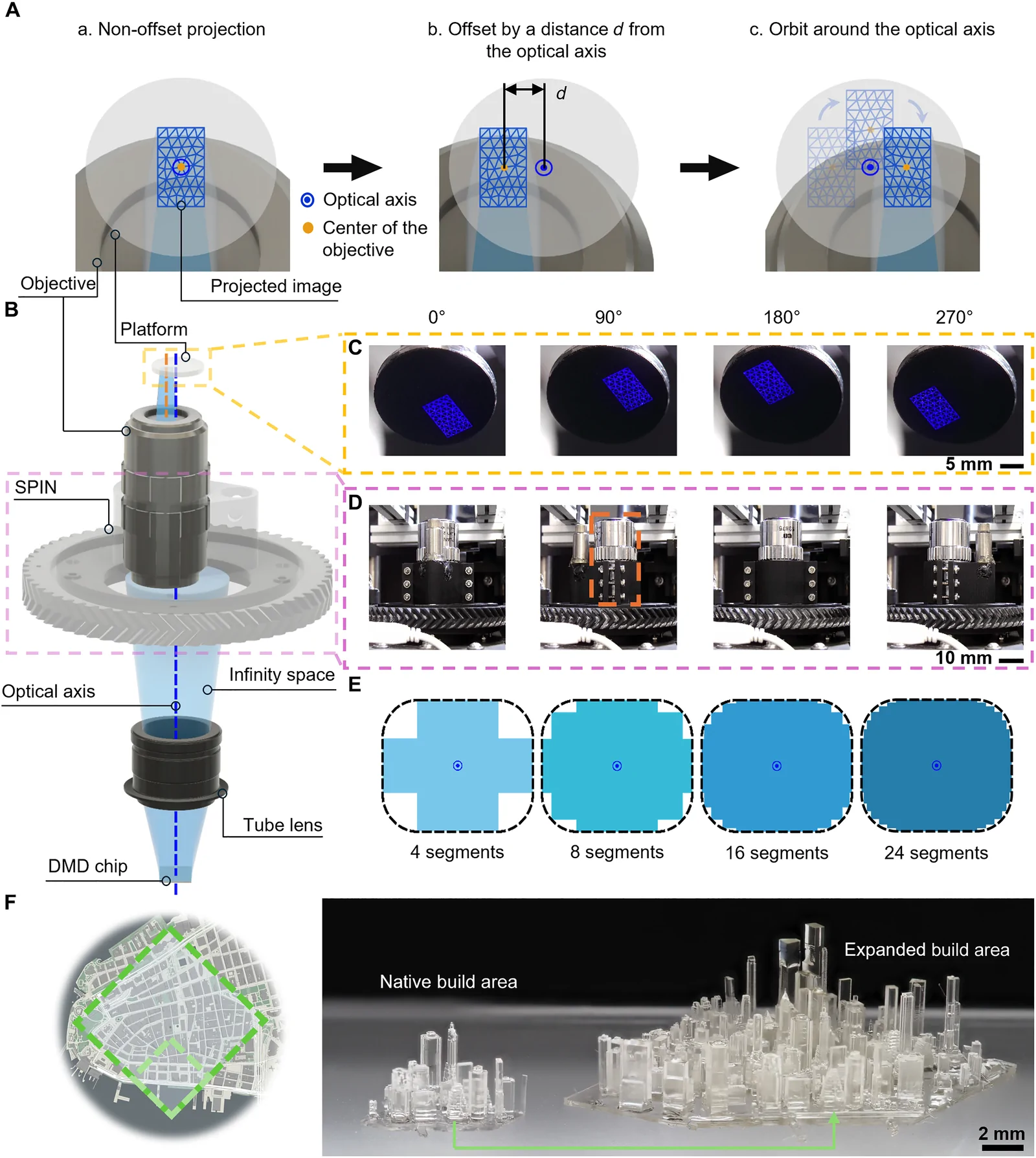
Methodology and mechanical design of the system for build area expansion. (**A**) Methodology for expanding the build area: (a) image projected at the center before installation of SPIN, (b) projected image laterally shifted by a distance d from the center, and (c) projected image orbiting about the center under SPIN control. (**B**) Schematic of the printer’s mechanical setup. (**C**) Projected images at four rotation angles: 0°, 90°, 180°, and 270°. (**D**) Photographs of the SPIN assembly at the corresponding rotation angles. The objective is indicated by the orange dashed box. (**E**) Build area coverage by tiling 4, 8, 16, and 24 segments. (**F**) Representative Manhattan skyline models printed using the native and expanded build areas. The downtown Manhattan STL model was purchased from MicroMisto on Cults. Scale bars, 5 mm (C), 10 mm (D), and 2 mm (F). Schematics in (A) and (B) were rendered using Autodesk Fusion 360.

Figure 1B presents a simplified schematic of the mechanical and optical architecture of the system. A 385-nm light-emitting diode (LED) is directed toward a DMD within a light engine. The DMD then reflects the light into the optical train using its array of 2560×1600 micromirrors, which also defines the resolution of the projected image. As the light passes through a tube lens, it becomes collimated and creates a light field that is commonly referred to as the infinity space. For conventional microscope-based projection optics, an infinity-corrected objective is placed at a calculated position along the optical axis to refocus the light onto the build platform at a set magnification. Herein, the objective is purposely offset relative to the primary optical axis to shift the projected image while still capturing a substantial portion of rays emanating from the tube lens, allowing the projected image to maintain its spatial resolution. Further details on optical simulations are discussed in the Optomechanical simulation and characterization section.

As shown in Fig. 1 (C and D), the patterned 2D image, at the projector’s native aspect ratio and resolution, is focused and projected onto the build platform. By orbiting the objective about the optical axis to 0°, 90°, 180°, and 270°, the image follows the motion and is projected at the corresponding angular position, as demonstrated. The projected image dimensions, resolution, and cumulative swept area primarily depend on the DMD mirror count and aspect ratio, optical magnification, and objective shifted distance. In our design, an average shifted distance d of 4.8 mm combined with a projected pixel size measured to be 3.86 μm increases the printable area from 60.4 to 290.5 mm^2^, corresponding to a 4.8-fold area expansion.

During printing, each layer is fabricated through a sequential exposure process. The printer first projects one image segment and then projects a dark frame while SPIN rotates the objective to the next angular position. The next image segment is then projected, and this sequence is repeated until the full layer is covered. Additional details of the methodology are provided in the Fabrication strategies section. Depending on the size and geometry of the part being printed, a finite set of angular segments can be selected to ensure sufficient coverage of the full build area. To demonstrate this, Fig. 1E shows coverage for 4, 8, 16, and 24 segments, spaced at equal angular increments across the build area, which cover 75, 91, 96, and 98% of the expanded build area, respectively. Infinite segments would be needed to reach 100% of the expanded build area, but to reduce operational complexity, the objective does not need to visit every possible defined position within the expanded build area. This approach effectively reduces computational expenses and increases printing efficiency. By shifting the objective further, a hole can be formed in the middle of the build plane such that a 2D annular region projection build area is produced. Although for most general use cases, it is preferable to have the center of the build plane covered by at least one projection, this annular configuration would be intrinsically beneficial for fabricating specialty structures like latticed tubes or stents. A full derivation of the relevant calculations is presented in the Supplementary Materials (section S5).

The system additionally incorporates CLIP by replacing the standard vat window with a tensioned oxygen-permeable Teflon film, which creates a nonphotopolymerizable dead zone at the bottom of the vat. This dead zone substantially reduces adhesion forces between the printed part and the film, thereby enabling both high printing speeds and reliable fabrication of fine microstructures (*15*). To demonstrate the ability to expand build area while preserving feature size fidelity, a 3D model of the Manhattan skyline was successfully printed and expanded from ∼10 buildings within the native build area to more than 50 buildings within the expanded build area (Fig. 1F).

### Optomechanical simulation and characterization

The projection optics of this system integrate the principles of tilt-shift lens design into conventional projection-based VP to achieve an expanded build area. A representative optical simulation using relay lenses was performed to compare on-axis (Fig. 2Aa) and off-axis (Fig. 2Ba) objective configurations. As shown in Fig. 2, a small lateral shift of +5 mm (Fig. 2Bb) maintains a very similar point spread function compared to the native (unshifted) system (Fig. 2Ab). However, the shape of the point spread function is altered slightly. The native projection has a circularly symmetric spot, whereas the shifted projection becomes oblong along the *x* axis and maintains symmetry along the *y* axis. In addition, the shift introduces an asymmetry into the vignetting pattern and the modulation transfer function (MTF). The native (unshifted) system presented in Fig. 2Ac maintains a typical relative illumination profile with brightest illumination in the center with a falloff toward the edges. In contrast, the shifted system presented in Fig. 2Bc relocates the brightest region to approximately two-thirds of the way into the field of view. This behavior qualitatively agrees with experimental measurements (Fig. 2Ca). To establish baseline system performance and compensate for off-axis vignetting, grayscale intensity correction was applied as a system-specific calibration tool to normalize spatial illumination across the expanded field. 32 intensity correction masks were generated across two angular segment sets (spacings of 22.5° and 30°), covering ∼98% of the build area. Implemented via pulse-width modulation (PWM) with an 8-bit intensity resolution (values from 0 to 255), this spatial mapping ensures uniform exposure irradiance across the unique optical configuration of the printer.

**Fig. 2.**
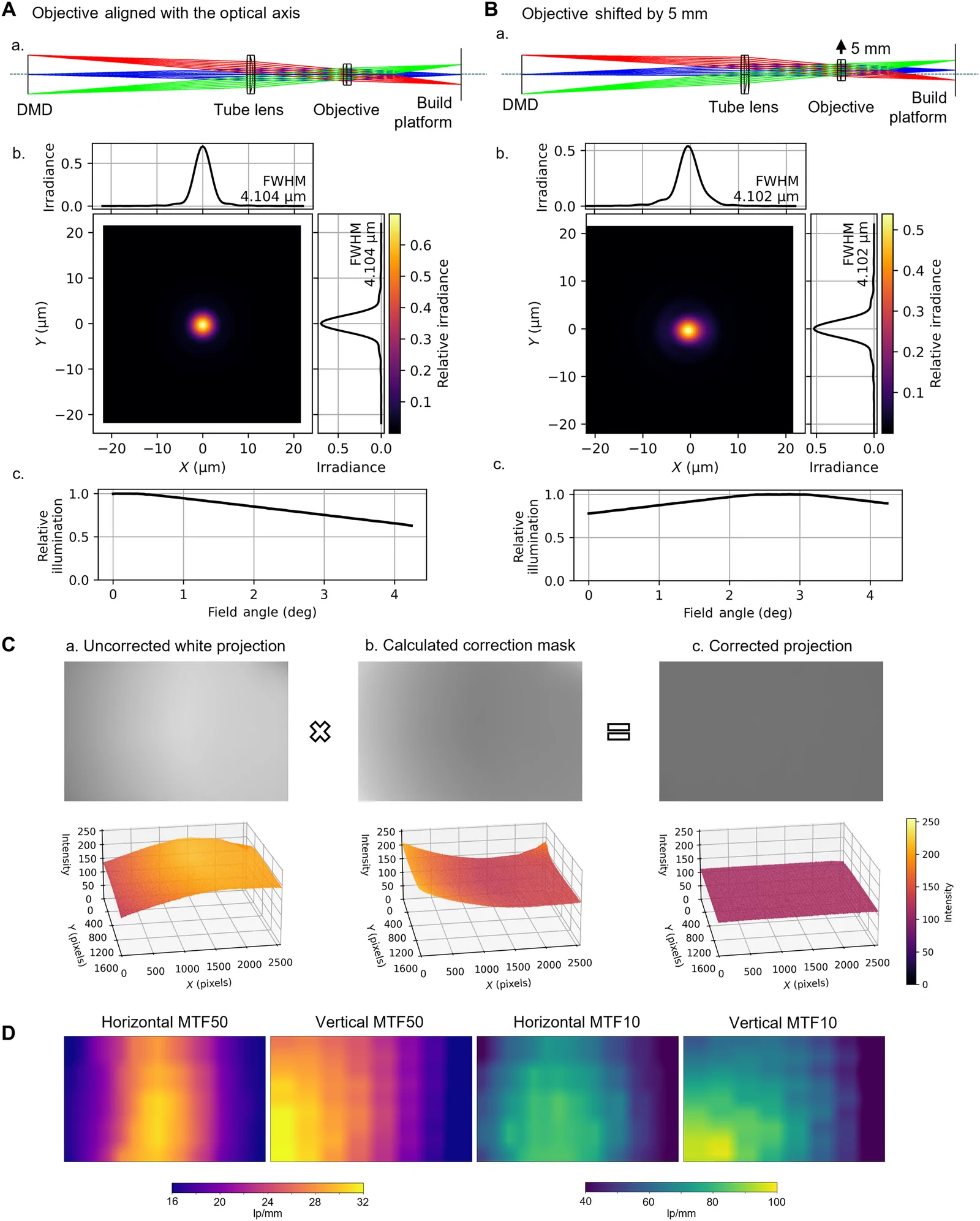
Optical simulation results and uniform intensity correction. (**A** and **B**) (a) Simulated ray diagrams, (b) corresponding point spread functions, and (c) vignetting intensity maps for the unshifted configuration (A) and shifted configuration (B). FWHM, full width at half maximum; deg, degrees. (**C**) Representative grayscale images and corresponding intensity contour plots at 180° rotation of (a) the experimentally captured uncorrected projection, (b) the calculated vignetting correction mask, and (c) the experimentally corrected projection. (**D**) Horizontal and vertical MTF50 and MTF10 spatial distributions across the corrected projection field at 180° rotation.

To generate the intensity correction masks, a uniform white image without correction was first projected onto the build plane and captured using a Thorlabs ultraviolet (UV) camera. The captured image provided a spatial intensity map $I_{c}\left(x,y\right)$ (Fig. 2Ca). The uncorrected intensity distribution was then normalized according to

$$I_{u}\left(x,y\right)=I_{c}\left(x,y\right)\frac{255}{\text{max}\left[I_{c}\left(x,y\right)\right]}$$ (1)

ensuring the maximum measured intensity reached the full dynamic range. The intensity correction mask was subsequently calculated as

$$I_{\text{correction}}\left(x,y\right)=255\frac{I_{\text{min}}}{I_{u}\left(x,y\right)}$$ (2)

where $I_{\text{min}}$ denotes the global minimum intensity across all 32 images (Fig. 2Cb). To preserve sufficient intensity resolution after correction, $I_{\text{min}}$ is defined as the minimum value within the lowest 99% of global intensity measurements. When the correction mask is applied during projection, the resulting image exhibits a substantially more uniform intensity distribution across the entire projection field (Fig. 2Cc). Additional images acquired at different angles are shown in fig. S6. In addition to vignetting, barrel distortion was observed and corrected using the Brown-Conrady model, and all correction procedures were incorporated into the slicing algorithm, as described in the following section.

To further evaluate optical performance, the MTF was experimentally measured across the projected field using a slant-edge method, as a function of field position and objective rotation (see section S7 for details). As shown in Fig. 2D, shifting introduces a spatially asymmetric MTF profile. Local MTF50 values span from 16 to 32 lp/mm across a single projection field, whereas limiting resolution of MTF10 ranges from 40 to 100 lp/mm. MTF characterization across the rotation angles (fig. S8) demonstrates that this asymmetric resolution pattern follows rotation of the objective.

### 3D CAD segmentation algorithm

We developed and implemented a segmentation algorithm in MATLAB that processes 3D computer-aided design (CAD) geometry by dissecting it into designated projection regions to describe the larger fabrication area. As illustrated in Fig. 3A, a Stanford Bunny model is used to demonstrate the slicing algorithm. First, the Bunny is sliced into a series of cross sections with a constant layer height, h. Each cross section is then subdivided into multiple projection regions, $\Omega_{1},\cdots ,\Omega_{m}$, where m denotes the number of regions per layer. Subsequently, each projection region is further partitioned into segments, $\sigma_{1},\cdots ,\sigma_{n}$, where n denotes the number of segments per layer (Fig. 3, B and C). The algorithm allows individual projection segments to be modified and defines the projection sequencing as described in the Fabrication strategies section.

**Fig. 3.**
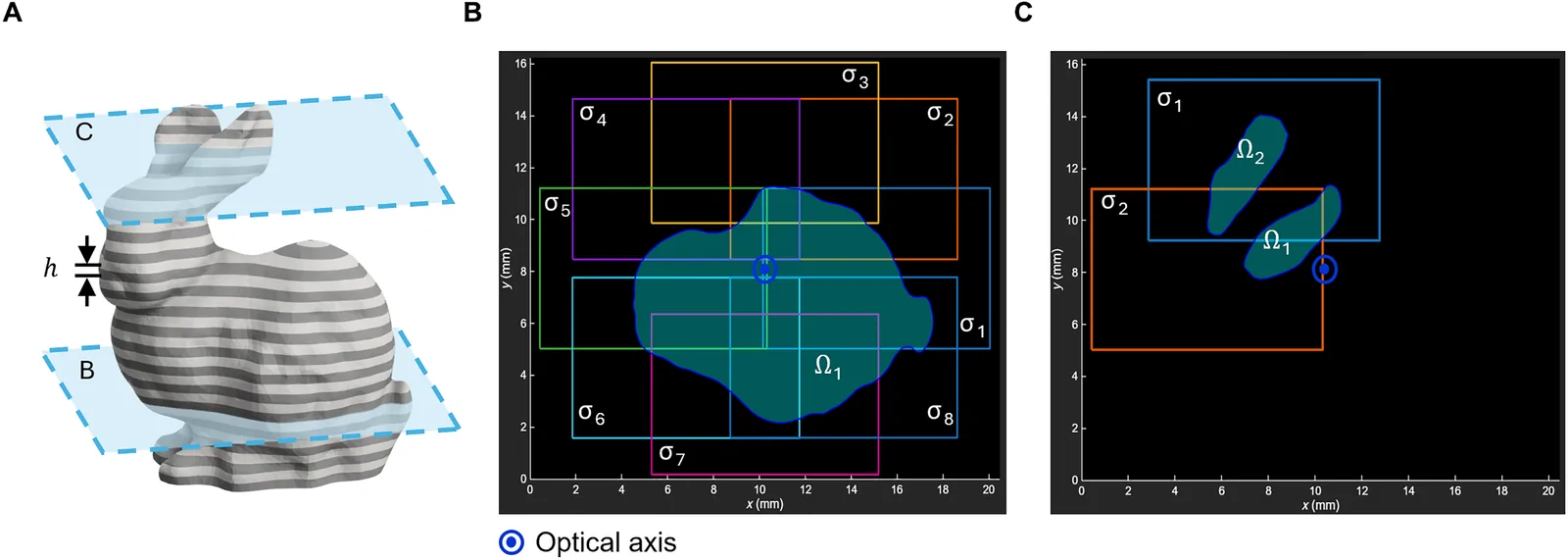
Image segmentation algorithm of the Stanford Bunny model. (**A**) The Stanford Bunny is sliced into layers with a constant layer height, h, visualized as alternating gray bands across the model. The Stanford Bunny image was rendered in Autodesk Fusion 360. (**B**) The algorithm generates multiple segments when slicing a large projection region. (**C**) The algorithm dynamically selects fewer segments when slicing smaller projection regions.

In cases where segments overlap (Fig. 3B), the shared exposed areas may be assigned grayscale values. These values are computed by dividing the initial areal intensity, $I_{\text{init}}$, by the number of overlapping segments, c, sharing a region Ω. The resulting intensity in the shared region is given by $I_{\text{init}}=\sum_{i=1}^{c}I_{i}$, where each $I_{i}=\frac{I_{\text{init}}}{c}$ represents the adjusted intensity contribution of region Ω within segment $\sigma_{i}$. A gain factor is included to account for curing nonlinearities. Initial studies show a positive correlation between the number of contributing exposures and the required total gain, warranting further study beyond the scope of this work.

In cases where the total cross-sectional contour area at a given slice height h can be fully described by fewer segments than initially assigned, the algorithm may perform dynamic segmentation (Fig. 3C). At each slice height, a minimal subset of segments, $\left\{\sigma_{1},\cdots ,\sigma_{k}\right\}\subseteq \left\{\sigma_{1},\cdots ,\sigma_{n}\right\}$, is selected such that the union of their corresponding projection regions sufficiently covers the required cross-sectional contour areas, denoted $\Omega_{\text{contour}}\left(h\right)$. This selection process is optimized using an integer linear programming (ILP) algorithm (*26*), where the objective is to minimize the number of segments needed to fully cover all contour segments. The ILP optimization defines a binary decision variable for each segment, indicating its inclusion in the subset. Constraints ensure that every point in $\Omega_{\text{contour}}\left(h\right)$ is covered by at least one segment. The ILP solver then identifies the combination of segments that achieves this coverage with the fewest segments. This dynamic segmentation approach reduces the number of projection segments per layer, thereby minimizing the number of rotations required during fabrication, if enabled. Consequently, the total fabrication time is decreased without compromising spatial coverage or resolution. In addition, this method provides greater flexibility and sensitivity in the assignment of parameters $I_{i}\left(h\right)$, enhancing the efficiency and scalability of the fabrication process.

Antialiasing may be performed on contour edges to improve intended shape fidelity. Antialiasing helps smooth the edges of contours, reducing visual artifacts that can occur due to the discrete nature of pixel-based representations. In the script, antialiasing is implemented using Gaussian filtering, which smooths contour edges by averaging pixel values in a local neighborhood. This process enhances the accuracy of the projected shapes, ensuring that the fabricated object closely matches the intended design.

This program additionally incorporates several functions designed to improve printing speed, accuracy, and reproducibility. To increase printing speed, the software accepts the resin’s working curve (*27*) as input and uses it to calculate the required printing parameters, including exposure time, non–exposure time, and stage velocity and acceleration, which are then written directly into the printing script. Printing accuracy is improved by allowing the objective shift distance to be defined for segments at different angular positions, compensating for mechanical misalignment in the system. To support reproducibility, the software provides an interactive preview of the segmented images at each layer before export, allowing users to quickly adjust segment number and placement before committing to the final sliced images. Together with the aberration correction methods described in the Optomechanical simulation and characterization section, the software enables accurate image projection with uniform intensity and facilitates rapid iteration and correction of configuration errors, contributing to a robust and reproducible printing workflow.

### Fabrication strategies

Given a rotary segmented cross-sectional approach, several different modalities of sequencing these segments arise, each with potential trade-offs in speed, complexity, and secondary outcomes. Fabrication modes may make use of a variety of mechanical, optical, and software parameters orchestrated in a multitude of timeframes. Potential parameters enabled herein to be modulated may include, but are not necessarily limited to objective rotation, *z*-stage translation, *z*-stage pumping, LED intensity or PWM variation, exposure time, dark time, and coincident timing. Here, four fabrication strategies are presented, using the Stanford Bunny model to demonstrate the fabrication process for a single layer.

Single projection mode (Fig. 4A): When the projection region falls entirely within a single segment, the fabrication method defaults to conventional VP operation. Within each layer, a single projection segment, σ, is used to expose the corresponding projection region(s), $\Omega_{i},$ curing the resin across the entire layer height, h. The region $\Omega_{i}$ must not exceed the area defined by σ. This is followed by a *z*-stage translation to the next height, h+Δh, and a brief pause to allow resin to reach quiescence before the next exposure. The process is repeated sequentially until the entire object is fabricated. The areal intensity $I_{i}$ for each σ may be dynamically adjusted based on factors such as cumulative exposure, layer height, and desired fabrication speed (*16*, *28*, *29*).

**Fig. 4.**
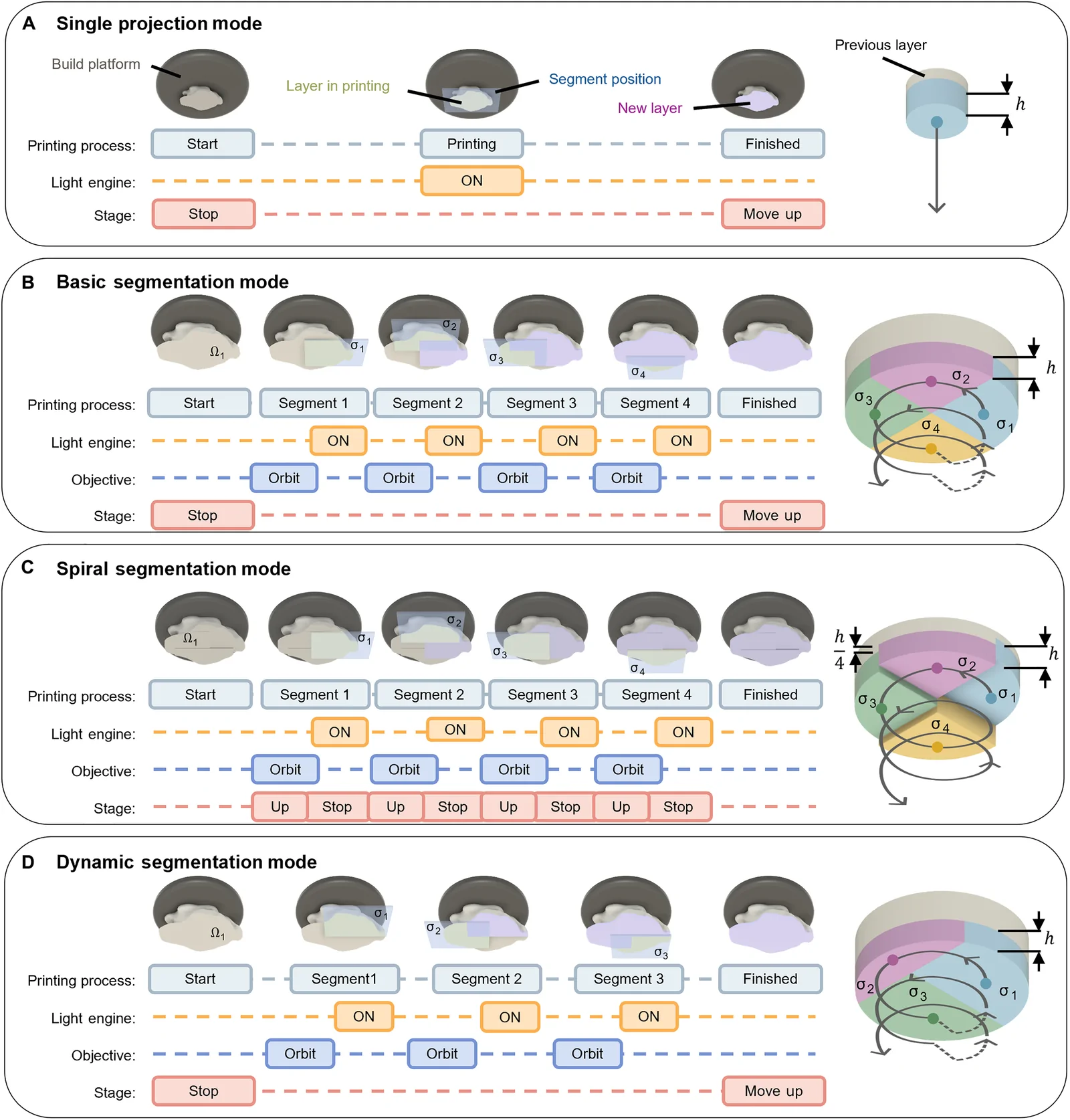
Fabrication strategies for expanding the build area. (**A**) Single projection mode, in which the full object cross section is exposed within one native projection area. (**B**) Basic segmentation mode, in which each layer is divided into multiple segments that are sequentially projected as the objective rotates between positions. (**C**) Spiral segmentation mode, in which objective rotation is coupled with incremental *z*-stage motion to distribute segmented exposures along a helical printing path. (**D**) Dynamic segmentation mode, in which only the required angular segments are projected for each layer to reduce unnecessary exposures and improve printing efficiency. Images were rendered from the CAD model in Autodesk Fusion 360.

Basic segmentation mode (Fig. 4B): For cases where the projection region exceeds the size of a single segment, each cross section is divided into multiple projection segments $\sigma_{1},\cdots ,\sigma_{n}$, which are separated by an equal angular increment $\Delta \varphi =\frac{360{}^{\circ}}{n}$. Within each layer at height h, the projection system sequentially exposes each segment $\sigma_{i}$. After each exposure, the projection is blanked (i.e., a fully black image), and the objective orbits about the optical axis by Δφ to the next angular position corresponding to $\sigma_{i+1}$. This process repeats until all n segments have been projected at height h. The objective then returns to its initial orientation, the *z*-stage translates to the next height h+Δh, and the sequence begins again with $\sigma_{1}$. This pattern is repeated until the entire object is fabricated. In this mode, the system stops and projects at every segment position for each layer, regardless of whether a given $\sigma_{i}$ contains a portion of the object’s geometry or is geometrically redundant.

Spiral segmentation mode (Fig. 4C): This mode builds on the basic segmentation mode by using the same projection and objective translation sequence but introduces incremental *z*-stage translation between segments. Instead of exposing all n angular segments at a fixed layer height h, each layer is subdivided into n sublayers of height h/n, corresponding to n angular segment positions $\sigma_{1},\cdots ,\sigma_{n}$. After each segment $\sigma_{i}$ is projected, the projection is blanked, the objective orbits by $\Delta \varphi =\frac{360{}^{\circ}}{n}$, and the *z*-stage translates upward by h/n. This continuous sequence forms a helical exposure path through the resin and printed parts. By distributing exposure more continuously along the *z* axis, spiral segmentation mode reduces abrupt layer transitions, improves surface smoothness, and enhances continuity between adjacent segment exposures.

Dynamic segmentation mode (Fig. 4D): The projection system fabricates each layer using only the minimum subset of projection segments $\left\{\sigma_{1},\cdots ,\sigma_{k}\right\}\subseteq \left\{\sigma_{1},\cdots ,\sigma_{n}\right\}$ identified by the dynamic segmentation algorithm as necessary to describe the object’s geometry at height h. For each required segment $\sigma_{i}$, the system exposes the corresponding region $\Omega_{i}\left(h\right)$, then blanks the projection and orbits the objective to the angular position associated with the next required segment. These angular positions do not necessarily correspond to sequential multiples of Δφ, depending on the geometry of the layer. After all required segments for a given layer are projected, the objective orbits to the first required segment of the next layer, the *z*-stage translates to height h+Δh, and the process repeats. This continues until the entire object is fabricated, with the system stopping only at the minimum number of segment positions needed to describe the geometry at each layer. This mode substantially increases printing speed by avoiding unnecessary segment positions, particularly for tapering geometries like the example shown in Fig. 3C, which require fewer projections than the midsection.

### Experiment validation demonstration

To demonstrate expansion of the build area while achieving sub–50 μm features, we fabricated several structures under distinct fabrication modes (movies S1 to S3; see section S8 for additional details). In Fig. 5A, four Stanford Bunny models are printed using trimethylolpropane triacrylate (TMPTA) to compare a single projection native build area against basic, dynamic, and spiral modes with layer height of 16 μm. All three rotary modes produce parts with an area expansion of sixfold relative to the native area print, with comparable morphology across modes. However, the differing methodologies introduce distinct improvements. Basic mode expands the printable area by stitching together sequential projections at fixed angular positions. With the default configuration of 16 angular segments spaced by 22.5°, spiral mode separates each 16-μm layer into 16 sublayers of 1 μm, leading to smoother layer transitions and enhanced interlayer adhesion in the printed part. In contrast, the dynamic mode prioritizes speed by skipping unnecessary angular segments, reducing the overall print time by approximately threefold compared with the basic and spiral modes (movie S1).

**Fig. 5.**
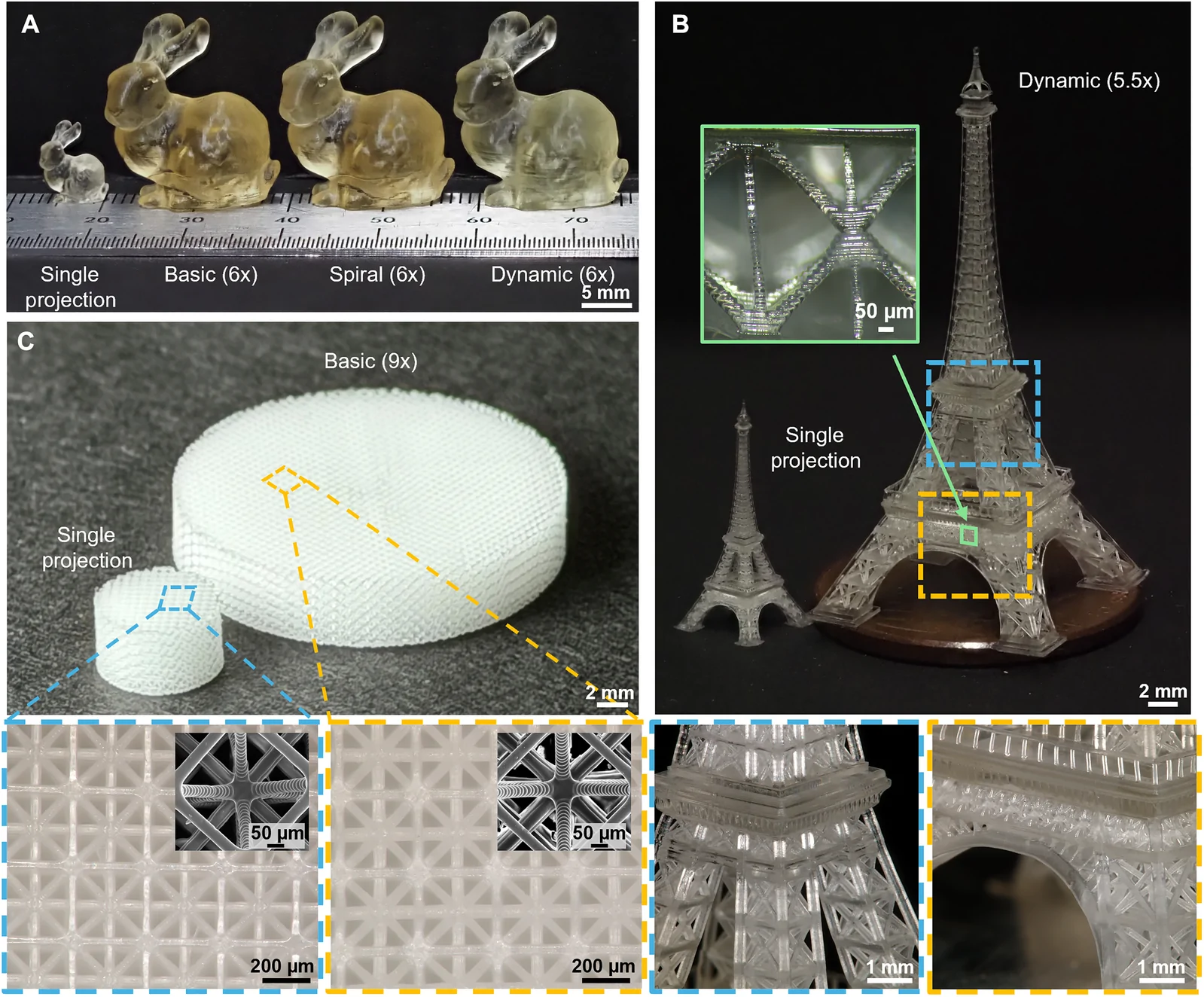
Representative printing results. (**A**) Stanford Bunny models printed in single projection and in basic, spiral, and dynamic modes, each corresponding to a sixfold area expansion. (**B**) Eiffel Tower models printed in single projection and dynamic modes, corresponding to a 5.5-fold area expansion. The green inset shows a high-magnification view of a printed lattice feature, and the blue and orange dashed boxes indicate regions shown in the corresponding magnified views below. (**C**) Octet lattices printed in single projection and basic mode, corresponding to a ninefold area expansion, with a 50-μm strut thickness and 500-μm unit cell. Blue and orange dashed boxes indicate the regions shown in the corresponding magnified views. SEM images of representative unit cells are shown as insets. Scale bars, 5 mm (A); 2 mm [(B), main image], 50 μm [(B), inset], and 1 mm [(B), magnified views]; and 2 mm [(C), main image], 200 μm [(C), magnified views], and 50 μm [(C), SEM insets].

Figure 5B compares Eiffel Tower structures printed with PR48 using single projection and dynamic segmentation modes. Dynamic segmentation enabled the Eiffel Tower model to achieve a 5.5-fold increase in cross-sectional area and a 13-fold increase in printed volume while preserving fine features, including a resolved truss strut thickness of 30 μm. Last, octet lattice disks with 50-μm-diameter struts and a 500-μm unit cell were printed in PR48 using both single projection and basic modes (Fig. 5C). The largest lattice compatible with the native projection area measures 5 mm in diameter, whereas the expanded area enables a lattice measuring 15 mm in diameter. This corresponds to a 3.2-fold increase in diameter and an overall ninefold expansion in the projected area. Within the larger lattice, most unit cells are printed with the designed strut diameters of 50 μm, as shown in the magnified views and in section S9, which provides a top-view image of the entire lattice. These feature sizes can be isotropically reduced to ≤10 μm through pyrolysis to form carbon architectures while maintaining practical macroscopic form factors, making them suitable for electrochemical applications such as scalable battery and reactor component production (*30*).

## DISCUSSION

This work introduced a cost-effective strategy for expanding the build area while preserving the native projected pixel size of a VP 3D printer. By orbiting the objective in a circular path around the optical axis through SPIN, the build area expands from 60.4 to 290.5 mm^2^ (4.8-fold) at a projected pixel size of 3.86 μm. Note that the average shifted distance was 4.8 mm, which was chosen to demonstrate effective area expansion within a practical mechanical and optical range. For this system, using the same pixel size and numbers of pixels, the critical maximum offset that maintains continuous coverage of the build platform is 5.83 mm, yielding a build area of 355.4 mm^2^ (5.9-fold larger than the native area). Additional details of this calculation are provided in section S5. At larger shifted distances, vignetting becomes increasingly pronounced. Restoring intensity uniformity through vignetting correction reduces the effective intensity resolution available per pixel in highly corrected regions. However, printing fidelity is not determined by effective intensity resolution alone and can be maintained by optimizing exposure time, projected intensity, resin response, and overall intensity uniformity.

We additionally performed optical simulations to compare shifted and unshifted projection configurations, evaluating the optical train response, point spread functions, and relative illumination as a function of field angle. In parallel, we implemented an experimental workflow for intensity uniformity correction and aberration compensation using a Thorlabs UV camera. These aberration corrections enable even illumination across the entire build area.

To enable printing across the enlarged field, we developed a segmentation algorithm that allows multiple fabrication modes, including single projection, basic segmentation, spiral segmentation, and dynamic segmentation. In single projection mode, the system exposes the entire layer’s 2D contour in one projection, but the build area is limited to the native projection field. In contrast, the basic segmentation mode partitions each layer’s contour into multiple segments, which are sequentially projected during printing to achieve build area expansion. Spiral segmentation mode, built on basic segmentation mode, improves interlayer adhesion by introducing small, controlled variations in curing depth across segments within a layer. Dynamic segmentation mode further accelerates printing by skipping segments with no content or with only overlapping regions. All these functionalities have been fully implemented in the system, with representative printed results of the Stanford Bunny shown in Fig. 5A. Additional structures, such as Eiffel Tower and an octet lattice, further validate the capability of this work to achieve both a large build area and fine structural resolution.

It should be noted that, although the CLIP was used in this demonstration of the technique, it is not strictly necessary. This method could be adapted to any VP-based or neighboring technique that uses similar projection optics, thereby broadening its applicability and potential impact.

### Optical design

In an infinity-corrected microscope system, lateral displacement of the objective relative to the tube lens, or vice versa, does not inherently degrade image quality if the optical train remains within its designed correction, field, and aperture limits. For sufficiently small lateral shifts, the marginal rays remain within the clear aperture and continue to propagate through regions of the optics where aberrations are acceptably corrected. Under ideal conditions, the displacement primarily translates the ray bundle laterally rather than fundamentally changing its convergence, numerical aperture, or imaging quality.

Lateral shifting can and does introduce vignetting when the displacement approaches or exceeds the designed clear aperture or image circle of either component. In this regime, portions of the collimated beam are clipped by mechanical mounts, lens barrels, or internal stops. Such clipping reduces the light throughput and alters the point spread function. As seen in Fig. 2, vignetting manifests as a gradual falloff in illumination at the focal plane, or as asymmetric darkening when the beam is truncated more severely on one side. The presence of vignetting does not usually imply a loss of aberration correction. Instead, it reflects a geometric limitation in how much of the ray bundle can be transmitted. Thus, although moderate lateral shifts largely preserve image quality by simply translating the ray bundle, excessive shifts that induce vignetting can compromise brightness, field uniformity, and quantitative imaging performance even when the optical corrections remain nominally intact.

To evaluate the spatial frequency response of the system, the MTF was experimentally characterized using a slanted-edge MTF method by projecting a perfect-edge target from the DMD directly onto a charge-coupled device (CCD) camera placed at the focal plane (fig. S7). The target edges projected by the DMD were perfectly horizontal and vertical relative to the DMD pixel grid, ensuring that the projected features were defined by clean, rectilinear pixel boundaries. Subpixel sampling for the slanted-edge analysis was instead achieved by physically rotating the CCD camera by ∼5° about its optical axis, as indicated in fig. S7A. For each target, the left, right, top, and bottom edges were independently cropped and analyzed to extract localized horizontal and vertical MTF values (fig. S7B). The edge spread function was reconstructed via denoised subpixel sampling along the slanted edge, differentiated to obtain the line spread function, and Fourier transformed to determine the MTF. MTF50 and MTF10 values were then extracted for each field location and for each objective rotation angle.

The measured results characterize the spatial imaging performance of the shifted optical architecture across the projected field. As shown in Fig. 2D and fig. S8, the shifted optical path results in an asymmetric MTF profile that follows the rotation of the objective. Across the field, MTF50 values range from 16 to 32 lp/mm, which correspond to 31 to 15 μm feature widths. Resolution-limiting MTF10 values range from 40 to 100 lp/mm across a single projection field, corresponding to 12.5- to 5-μm minimum theoretically resolvable feature sizes. Furthermore, the best MTF performance was measured near 0° and 315°, whereas the reduced MTF performance was observed near 135° to 225°, indicating some orientation-dependent defocusing in the shifted optical path. The changes in MTF50 and MTF10 are gradual at rotation angles 0° to 45° and 270° to 315° and more abrupt at 90° to 225°, with horizontal and vertical heatmaps demonstrating similar spatial behavior across the MTF50 and MTF10 thresholds. This behavior is consistent with defocus from field-plane tilt, likely due to minor mechanical misalignment of either the CCD sensor or objective during assembly. Therefore, the lower MTF values at these angles should be interpreted as a conservative measurement of system performance under imperfect alignment. The system maintains measurable contrast across the entire sampled projection area, demonstrating that lateral objective shifting can expand the usable projection field while retaining sufficient resolution and contrast for microscale patterned exposure.

### Additional fabrication methods

Beyond the modes evaluated experimentally, the SPIN architecture is compatible with several additional optical and fabrication strategies, including objective swapping (*31*), spirograph objective movement paths, dual- or multiwavelength sources (*32*), resin injection (*33*, *34*), paralleled sources or optical expansion (*25*), shutters (*35*), sequential translation of the objective radially outward, and translation of the entire optical train relative to the stage and vat. Several potential modalities using our SPIN method are described herein. However, these represent a starting point rather than an exhaustive list.

#### Continuous rotation mode

The objective orbits continuously at a constant angular velocity, synchronized with the projector’s refresh rate, to expose a sequence of angular segments $\sigma_{1},\cdots ,\sigma_{n}$ without stopping. For instance, at a refresh rate of 120 Hz (as supported by the InVision Firebird), a 1-s objective sweep can expose n=120 segments, each corresponding to an angular increment of Δφ=3°. Increasing the sweep duration to 4 s improves angular resolution to Δφ=0.75°, enhancing the fidelity of the projected geometry. During each sweep, the system fully exposes the layer in a continuous motion. The *z*-stage remains stationary during the exposure sweep and translates upward Δh during the subsequent sweep (blanked projection), allowing the next layer to begin on the third sweep relative to the initial. This staggered timing ensures uninterrupted rotation while maintaining layer fidelity. In continuous rotation, these operational sweeps can incorporate cumulative exposure tracking, evaluating the integral of pixel intensity along the projection path, to more precisely dose the intended geometry. In addition, adopting rotational segmentation (i.e., binning along arc lengths as a function of refresh rate) may further improve geometric accuracy by aligning exposure with the curvature of the objective path.

#### Continuous spiral mode

The objective orbits continuously at a constant angular velocity while the *z*-stage simultaneously translates upward at a constant speed. The projected segments are synchronized with both the angular position of the objective and the vertical position of the stage, allowing the object to be fabricated along a continuous helical path.

#### Dynamic spiral mode

This mode combines dynamic and spiral segmentation modes. An object may be fabricated by stopping only at necessary segments as the *z*-stage sequentially climbs.

#### Dynamic layer segmentation

For cross sections of repeated geometry, the *z*-stage may be stepped in an increment consistent with the number of repeating cross sections and the exposure increased to the extrapolated value for the cumulative layer height.

#### Sequential, secondary sweep path

Sequential translation of the objective radially outward via an integrated translation mechanism within the objective’s rotational gear would result in a combination of the solid projection at a center-overlapping offset and an annular projection sweep path. This would result in up to a 14.7-fold build area expansion relative to a native 3.86-μm 2K DMD projection. This may also be achieved in some cases via two objectives built into the rotational gear each at a different, set offset from the optical axis along with a circular and annular shutter.

### Alignment and resolution characteristics

Precise alignment of optical assemblies is a central challenge in the development of experimental printers targeting sub–50 μm features. Even small misalignments can lead to layer-to-layer errors, geometric distortions, incomplete curing, or surface artifacts. During the initial assembly and testing of our system, several recurring defects are observed in localized regions, which were attributed to accumulated system tolerances. These defects arose from multiple error sources, including inaccurate homing of the rotary stage, mismatches between the shifted distances of projected images and their corresponding sliced images, and general optical misalignment. To address these sources of error, we implemented a set of calibration and alignment procedures, as described below.

SPIN uses an optical homing strategy based on a simple pair of red LED and photoresistor placed across the objective drive gear. During homing, an aperture in the gear passes through the optical path, allowing the controller to determine the objective’s 0° rotational reference. To evaluate homing and rotational repeatability, a diagonal grid pattern was projected onto a CCD camera sensor, and images were acquired after initial homing, after repeated homing, and after rotating the objective through multiple full revolutions, as described in section S11. Color overlay analysis showed strong overlap between the initial and repeated measurements, with misalignment within one CCD pixel, corresponding to 3.45 μm. This optical homing strategy supports reliable layer-to-layer registration in SPIN.

To minimize angular alignment errors during SPIN rotation, we measured the image shift at each rotation angle and applied the corresponding correction in the slicing script (see section S12 for details). A 100-pixel-diameter circle was projected onto a UV-sensitive CCD camera at 24 equally spaced angular positions over one full rotation. The shift distance at each angular position was calculated relative to the fitted rotational center using MATLAB. These offsets were then incorporated into the slicing script to compensate for misalignment caused by imperfections in the mechanical structure. The initial correction improved the overall alignment but left residual section-to-section misalignment. Therefore, the remaining angular offsets were further refined by experimentally measuring the projected box positions in ImageJ and updating the slicer with these correction values. This calibration substantially improved overlap between circular and grid calibration patterns across all angular positions, confirming accurate projection alignment during SPIN rotation.

The resolution and alignment performance of SPIN were further characterized using projected line patterns (see sections S13 to S15 for details). For a single projection segment, line patterns with designed widths of 7.7 to 50.2 μm, corresponding to 2 to 13 pixels, were projected and printed at 0°. Scanning electron microscopy (SEM) analysis showed printed linewidths of 9.8 ± 2.2, 12.4 ± 2.6, 20.9 ± 1.1, 31.7 ± 2.9, and 50.9 ± 2.7 μm for the 2-, 3-, 5-, 8-, and 13-pixel patterns, respectively (fig. S13). These results confirm the capability of the printer system to fabricate sub–50 μm microscale features. To assess projection performance across rotation angles, 2-pixel line patterns were also projected and captured by a CCD camera at rotation angles from 0° to 315° in 45° increments, with the line features remaining distinguishable at all tested angles (fig. S14). To evaluate alignment in overlapped projection regions, an 8-pixel line pattern was sliced into four overlapping segments and evaluated by both CCD imaging and printing on the build platform. CCD-captured images and SEM images of the printed overlapped regions showed clear and continuous line features, with measured alignment errors below 10 μm (fig. S15).

The remaining alignment error in the current prototype is likely associated with mechanical tolerances in the 3D printed structural components and rotary assembly. These factors can introduce runout, tilting, and small projection shifts during rotation. Future implementation with precisely machined components is expected to further improve mechanical stability, reduce residual alignment error, and enable finer feature resolution. The results presented here demonstrate that the proposed approach can expand the effective build area while preserving microscale feature fidelity, suggesting that further improvements are likely to arise from hardware refinement rather than changes to the underlying SPIN methodology.

## MATERIALS AND METHODS

### Rotary fabrication hardware design

Primary optical train components include a 385-nm DLP light engine (9000 Firebird Light Engine, In-Vision Technologies AG) modified to house a tube lens (54-774, Edmund Optics) with corresponding 2× infinity-corrected objective (46-142, Edmund Optics). The objective was laterally offset from the optical axis and orbited around it via a custom 3D printed gear setup driven by a NEMA 17 stepper motor (17HD48002-22B, Longruner). Motor control was achieved using an Arduino microcontroller (ELEGOO UNO R3) and a two-phase stepper driver (Cloudray DM422S). After vignetting correction, the light engine provided an adjustable intensity range of 6 to 152 mW/cm^2^ at the focal plane, measured using an 843-R power meter with a Model 818-SL sensor (Newport). A vibration-isolated optical table was used to minimize global alignment errors. A dual-axis digital angle protractor (DXL360S, Roeckle Neigungsmesstechnik e.K.) with a 0.01° resolution was used to set and verify angular alignment of the rotary components and the *z*-stage that carries the build platform. The tilt of the objective, the resin vat, and the build platform were held within 0.1° throughout rotation and across build positions. A Thorlabs Kiralux 12.3MP CMOS Compact Scientific Camera was used in objective shifted distance measurements and vignetting correction. Photopolymerizable resin was held in a machined aluminum vat with tensioned Teflon AF2400 (Carbon3D) film at the bottom. The print substrate was a Ø25.4-mm SEM mount (16144, TedPella), vertically translatable by a high-precision *z*-stage (GTS70V, Newport).

### Rotary fabrication software design—Printer control

The printer was primarily controlled by an updated CLIP3DGUI program, a custom C++ application built with Qt Creator. The interface allowed users to load images for projection, set default parameters, and define a print script specifying exposure time, dark time, LED intensity, stage velocity and acceleration, pumping height, and frame rotation angles. CLIP3DGUI directly coordinated the light engine and the translation stage, and it transmitted computed angles, rotational speed, and rotational acceleration to an Arduino microcontroller that drove the rotary system. The software executed the print workflow in a defined sequence, ensuring synchronized light delivery and motion control. In the current implementation, communication between the GUI and the microcontroller was unidirectional, with operational commands transmitted without real-time feedback. Incorporating bidirectional communication in future implementations would enable closed-loop control, further improving printing efficiency and system robustness.

### Rotary fabrication software design—CAD interpretation and rotary segmentation

An image segmentation software program was written in MATLAB (R2023a-R2025b) with the use of the Image Processing and Optimization Toolboxes (*26*, *36*). Generative AI platforms, including the OpenAI GPT family and the Gemini/Bard series accessed securely through the Stanford Medicine AI Sandbox enterprise environment, were used as development aids for code refinement, optimization, refactoring, and troubleshooting. All final programmatic structures, functional scripts, and interfacing components were audited, verified, and compiled by the authors. CAD was performed in SolidWorks 2023 and Autodesk Fusion 2024.

### STL models and attribution

The downtown Manhattan STL model was purchased from MicroMisto on Cults under a Cults Private Use license and used as a demonstration geometry. The Stanford Bunny model was downloaded from Thingiverse, where it was published by MakerBot under a Creative Commons Attribution 4.0 International license (CC BY 4.0). The Eiffel Tower model was obtained from the previously published work of Tumbleston *et al.* (*15*). Lattice structures were designed by the authors using nTop and Autodesk Fusion 2024. Third-party models were used only as demonstration geometries and are not claimed as original model designs by the authors.

### Material preparation and formulation

LOCTITE 3843 photopolymer resin (Henkel Corporation, Irvine, CA, USA) was used to fabricate structural components of the structure of this work. For experimental prints, a custom acrylate resin was formulated as previously described comprising 97.2 wt % TMPTA, 2.5 wt % diphenyl(2,4,6-trimethylbenzoyl)phosphine oxide (TPO) as the photoinitiator, and 0.3 wt % BLS1326. The working curve and rheological properties of this resin formulation have been previously characterized by Kronenfeld *et al.* (*37*) and Hsiao *et al.* (*16*), respectively. EPU Pro 50 Black was purchased from Carbon 3D (CA, USA). PR48 was purchased from CPS Polymers (Arkema, Inc, CO, USA).

### Analysis

Optical imaging was performed on an Olympus DSX1000 (Evident Scientific) with the corresponding analysis software. Images were analyzed on ImageJ/Fiji (*38*) and visualized in InkScape 1.4. Statistical analysis and visualization were performed using GraphPad Prism 10.5.0.

## Supplementary Materials

### The PDF file includes:

Sections S1 to S16 Figs. S1 to S16 Tables S1 to S3 Legends for movies S1 to S3 References

## References

[1] F. Alam, N. El-Atab, Multi-material 4D printing employing stimuli-responsive polymer composites using vat photopolymerization. Virtual Phys. Prototyp. 20, e2444576 (2025).

[2] X. Kuang, J. Wu, K. Chen, Z. Zhao, Z. Ding, F. Hu, D. Fang, H. J. Qi, Grayscale digital light processing 3D printing for highly functionally graded materials. Sci. Adv. 5, eaav5790 (2019). 10.1126/sciadv.aav5790 PMC6499595 31058222

[3] H. Cui, D. Yao, R. Hensleigh, H. Lu, A. Calderon, Z. Xu, S. Davaria, Z. Wang, P. Mercier, P. Tarazaga, X. R. Zheng, Design and printing of proprioceptive three-dimensional architected robotic metamaterials. Science 376, 1287–1293 (2022). 10.1126/science.abn0090 35709267

[4] E. Rossegger, R. Höller, K. Hrbinič, M. Sangermano, T. Griesser, S. Schlögl, 3D printing of soft magnetoactive devices with thiol-click photopolymer composites. Adv. Eng. Mater. 25, 2200749 (2023).

[5] Z. Zhu, D. Wang, M. Zhang, L. Dong, Q. Yu, C. Jiang, X. Zhu, M. Chen, K. Zhou, G. Gu, Multimaterial 3D printed soft robots with embedded actuation and sensing. Sci. Adv. 11, eadz2928 (2025). 10.1126/sciadv.adz2928 PMC12710710 41406224

[6] X. Hu, I. C. Yasa, Z. Ren, S. R. Goudu, H. Ceylan, W. Hu, M. Sitti, Magnetic soft micromachines made of linked microactuator networks. Sci. Adv. 7, eabe8436 (2021). 10.1126/sciadv.abe8436 PMC8177716 34088661

[7] S. M. Z. S. Bukhari, M. A. Bukhari, M. Hossain, Vat photopolymerization of smart polymers for biomedical applications. ACS Appl. Polym. Mater. 7, 12117–12144 (2025). 10.1021/acsapm.5c01487 PMC12481486 41036100

[8] Z. Wang, H. Kumar, Z. Tian, X. Jin, J. F. Holzman, F. Menard, K. Kim, Visible light photoinitiation of cell-adhesive gelatin methacryloyl hydrogels for stereolithography 3D bioprinting. ACS Appl. Mater. Interfaces 10, 26859–26869 (2018). 10.1021/acsami.8b06607 30024722

[9] Y. Bao, N. Paunović, J.-C. Leroux, Challenges and opportunities in 3D printing of biodegradable medical devices by emerging photopolymerization techniques. Adv. Funct. Mater. 32, 2109864 (2022).

[10] N. Rajesh, J. Hwang, Y. Xu, N. Esplin, C. S. Lowenhaupt, M. Hanabusa, M. E. Szafranski, R. K. S. Shinn, C. Huang, A. V. Fesenko, S. Zhang, P. G. Schorr, R. S. S. Shinn, J. M. Perry, J. M. DeSimone, 3D-printed latticed microneedle array patches for tunable and versatile intradermal delivery. Adv. Mater. 36, e2404606 (2024). 10.1002/adma.202404606 39221508

[11] Y. Er, O. Güler, G. Hekimoğlu, M. Nodehi, A. Ustaoğlu, A. Sarı, O. Gencel, T. Ozbakkaloglu, Thermophysical properties and solar thermal energy storage performance of phase change composites manufactured by vat photopolymerization 3D printing technique. J. Energy Storage 73, 109124 (2023).

[12] X. Xu, C. Guan, L. Xu, Y. H. Tan, D. Zhang, Y. Wang, H. Zhang, D. J. Blackwood, J. Wang, M. Li, J. Ding, Three dimensionally free-formable graphene foam with designed structures for energy and environmental applications. ACS Nano 14, 937–947 (2020). 10.1021/acsnano.9b08191 31891478

[13] H. Li, J. Dai, Z. Wang, H. Zheng, W. Li, M. Wang, F. Cheng, Digital light processing (DLP)-based (bio)printing strategies for tissue modeling and regeneration. Aggregate 4, e270 (2023).

[14] L. Wu, Y. Song, Recent innovations in interfacial strategies for DLP 3D printing process optimization. Mater. Horiz. 12, 401–417 (2025). 10.1039/d4mh01160k 39470616

[15] J. R. Tumbleston, D. Shirvanyants, N. Ermoshkin, R. Janusziewicz, A. R. Johnson, D. Kelly, K. Chen, R. Pinschmidt, J. P. Rolland, A. Ermoshkin, E. T. Samulski, J. M. DeSimone, Continuous liquid interface production of 3D objects. Science 347, 1349–1352 (2015). 10.1126/science.aaa2397 25780246

[16] K. Hsiao, B. J. Lee, T. Samuelsen, G. Lipkowitz, J. M. Kronenfeld, D. Ilyn, A. Shih, M. T. Dulay, L. Tate, E. S. G. Shaqfeh, J. M. DeSimone, Single-digit-micrometer-resolution continuous liquid interface production. Sci. Adv. 8, eabq2846 (2022). 10.1126/sciadv.abq2846 PMC9668307 36383664

[17] Y. Xu, H. Mao, C. Liu, Z. Du, W. Yan, Z. Yang, J. Partanen, Y. Chen, Hopping light vat photopolymerization for multiscale fabrication. Small 19, e2205784 (2023). 10.1002/smll.202205784 36541744

[18] M. P. Lee, G. J. T. Cooper, T. Hinkley, G. M. Gibson, M. J. Padgett, L. Cronin, Development of a 3D printer using scanning projection stereolithography. Sci. Rep. 5, 9875 (2015). 10.1038/srep09875 PMC4407727 25906401

[19] C. Vidler, K. Crozier, D. Collins, Ultra-resolution scalable microprinting. Microsyst. Nanoeng. 9, 67 (2023). 10.1038/s41378-023-00537-9 PMC10212948 37251709

[20] R. Yi, C. Wu, Y.-J. Liu, Y. He, C. C. L. Wang, Delta DLP 3-D printing of large models. IEEE Trans. Autom. Sci. Eng. 15, 1193–1204 (2018).

[21] Y. Wang, X. Li, S. Fan, X. Feng, K. Cao, Q. Ge, L. Gao, Y. Lu, Three-dimensional stretchable microelectronics by projection microstereolithography (PμSL). ACS Appl. Mater. Interfaces 13, 8901–8908 (2021). 10.1021/acsami.0c20162 33587597

[22] J. A. Jackson, M. C. Messner, N. A. Dudukovic, W. L. Smith, L. Bekker, B. Moran, A. M. Golobic, A. J. Pascall, E. B. Duoss, K. J. Loh, C. M. Spadaccini, Field responsive mechanical metamaterials. Sci. Adv. 4, eaau6419 (2018). 10.1126/sciadv.aau6419 PMC6286172 30539147

[23] N. A. Dudukovic, E. J. Fong, H. B. Gemeda, J. R. DeOtte, M. R. Cerón, B. D. Moran, J. T. Davis, S. E. Baker, E. B. Duoss, Cellular fluidics. Nature 595, 58–65 (2021). 10.1038/s41586-021-03603-2 34194019

[24] X. Zheng, W. Smith, J. Jackson, B. Moran, H. Cui, D. Chen, J. Ye, N. Fang, N. Rodriguez, T. Weisgraber, C. M. Spadaccini, Multiscale metallic metamaterials. Nat. Mater. 15, 1100–1106 (2016). 10.1038/nmat4694 27429209

[25] M. Kim, G. Yoo, B. Kim, Y. Song, B. J. Lee, Scalability enhancement in projection-based 3D printing through optical expansion. Addit. Manuf. 95, 104511 (2024).

[26] The MathWorks Inc., Optimization Toolbox User’s Guide, R2025b (2025); https://mathworks.com/help/optim/.

[27] P. F. Jacobs, *Rapid Prototyping & Manufacturing: Fundamentals of Stereolithography* (Society of Manufacturing Engineers, 1992).

[28] J. Bennett, Measuring UV curing parameters of commercial photopolymers used in additive manufacturing. Addit. Manuf. 18, 203–212 (2017). 10.1016/j.addma.2017.10.009 PMC5828039 29503807

[29] Z. D. Pritchard, M. P. de Beer, R. J. Whelan, T. F. Scott, M. A. Burns, Modeling and correcting cure-through in continuous stereolithographic 3D printing. Adv. Mater. Technol. 4, 1900700 (2019).

[30] P. R. Onffroy, S. Chiovoloni, H. L. Kuo, M. A. Saccone, J. Q. Lu, J. M. DeSimone, Opportunities at the intersection of 3D printed polymers and pyrolysis for the microfabrication of carbon-based energy materials. JACS Au 4, 3706–3726 (2024). 10.1021/jacsau.4c00555 PMC11522932 39483227

[31] Z. J. Geffert, Z. Xiong, J. Grutzmacher, M. Wilderman, A. Mohammadi, A. Filip, Z. Li, P. Soman, Multipath projection stereolithography for three-dimensional printing microfluidic devices. ACS Appl. Mater. Interfaces 16, 69807–69817 (2024). 10.1021/acsami.4c10547 PMC11660027 39626966

[32] K. C. H. Chin, G. Ovsepyan, A. J. Boydston, Multi-color dual wavelength vat photopolymerization 3D printing via spatially controlled acidity. Nat. Commun. 15, 3867 (2024). 10.1038/s41467-024-48159-7 PMC11078982 38719871

[33] I. A. Coates, W. Pan, M. A. Saccone, G. Lipkowitz, D. Ilyin, M. M. Driskill, M. T. Dulay, C. W. Frank, E. S. G. Shaqfeh, J. M. DeSimone, High-resolution stereolithography: Negative spaces enabled by control of fluid mechanics. Proc. Natl. Acad. Sci. U.S.A. 121, e2405382121 (2024). 10.1073/pnas.2405382121 PMC11406279 39231205

[34] G. Lipkowitz, T. Samuelsen, K. Hsiao, B. Lee, M. T. Dulay, I. Coates, H. Lin, W. Pan, G. Toth, L. Tate, E. S. G. Shaqfeh, J. M. DeSimone, Injection continuous liquid interface production of 3D objects. Sci. Adv. 8, eabq3917 (2022). 10.1126/sciadv.abq3917 PMC9519045 36170357

[35] M. S. Fischer, Cost-effective, open-source light shutters with Arduino control. HardwareX 19, e00548 (2024). 10.1016/j.ohx.2024.e00548 PMC11292492 39092421

[36] The MathWorks Inc., Image Processing Toolbox User’s Guide, R2025b (2025); https://mathworks.com/help/images/index.html.

[37] J. M. Kronenfeld, L. Rother, M. A. Saccone, M. T. Dulay, J. M. DeSimone, Roll-to-roll, high-resolution 3D printing of shape-specific particles. Nature 627, 306–312 (2024). 10.1038/s41586-024-07061-4 PMC10937373 38480965

[38] J. Schindelin, I. Arganda-Carreras, E. Frise, V. Kaynig, M. Longair, T. Pietzsch, S. Preibisch, C. Rueden, S. Saalfeld, B. Schmid, J.-Y. Tinevez, D. J. White, V. Hartenstein, K. Eliceiri, P. Tomancak, A. Cardona, Fiji: An open-source platform for biological-image analysis. Nat. Methods 9, 676–682 (2012). 10.1038/nmeth.2019 PMC3855844 22743772

[39] Carbon Inc., “EPU Pro 50 and 90 Black Technical Datasheet” (Carbon Inc., 2026).

