# Supplementary material for: Segmented projection image translation for large-area, high-resolution vat photopolymerization
Source: Sci Adv. 2026 Nov 11;12(46):eaef7779. doi: 10.1126/sciadv.aef7779 (PMC13753993; doi:10.1126/sciadv.aef7779)
Supplement: Supplementary file 1 [file sciadv.aef7779_sm.pdf]

Science Advances
AAAS

Supplementary Materials for

Segmented projection image translation for large-area, high-resolution vat photopolymerization

Jize Dai *et al.*

Corresponding author: Joseph M. DeSimone, jmdesimone@stanford.edu

Sci. Adv. **12**, eaef7779 (2026)
DOI: 10.1126/sciadv.aef7779

The PDF file includes:

Sections S1 to S16
Figs. S1 to S16
Tables S1 to S3
Legends for movies S1 to S3
References

Other Supplementary Material for this manuscript includes the following:

Movies S1 to S3

Supplementary Materials

1. Methods for expanding the build area in DLP printing

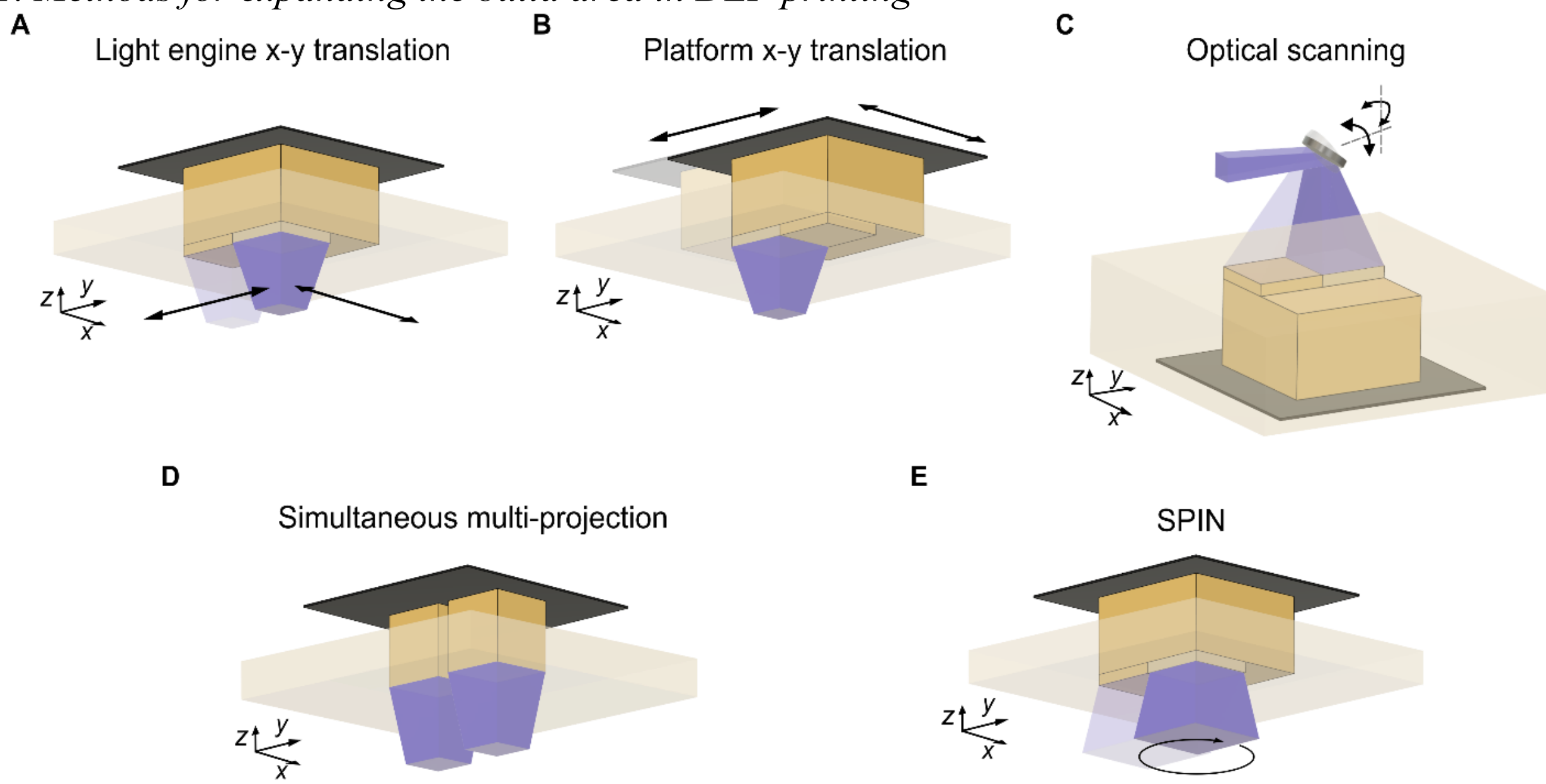

Fig. S1. Schematic illustration of five strategies for enlarging the build area in DLP printers. (A) translation of the light engine in the *xy*-plane, (B) translation of the build platform in the *xy*-plane, (C) optical scanning, (D) simultaneous multi-projection, and (E) segmented projection image translation (SPIN). All schematics were rendered using Autodesk Fusion 360.

2. Experimental setup of the printer

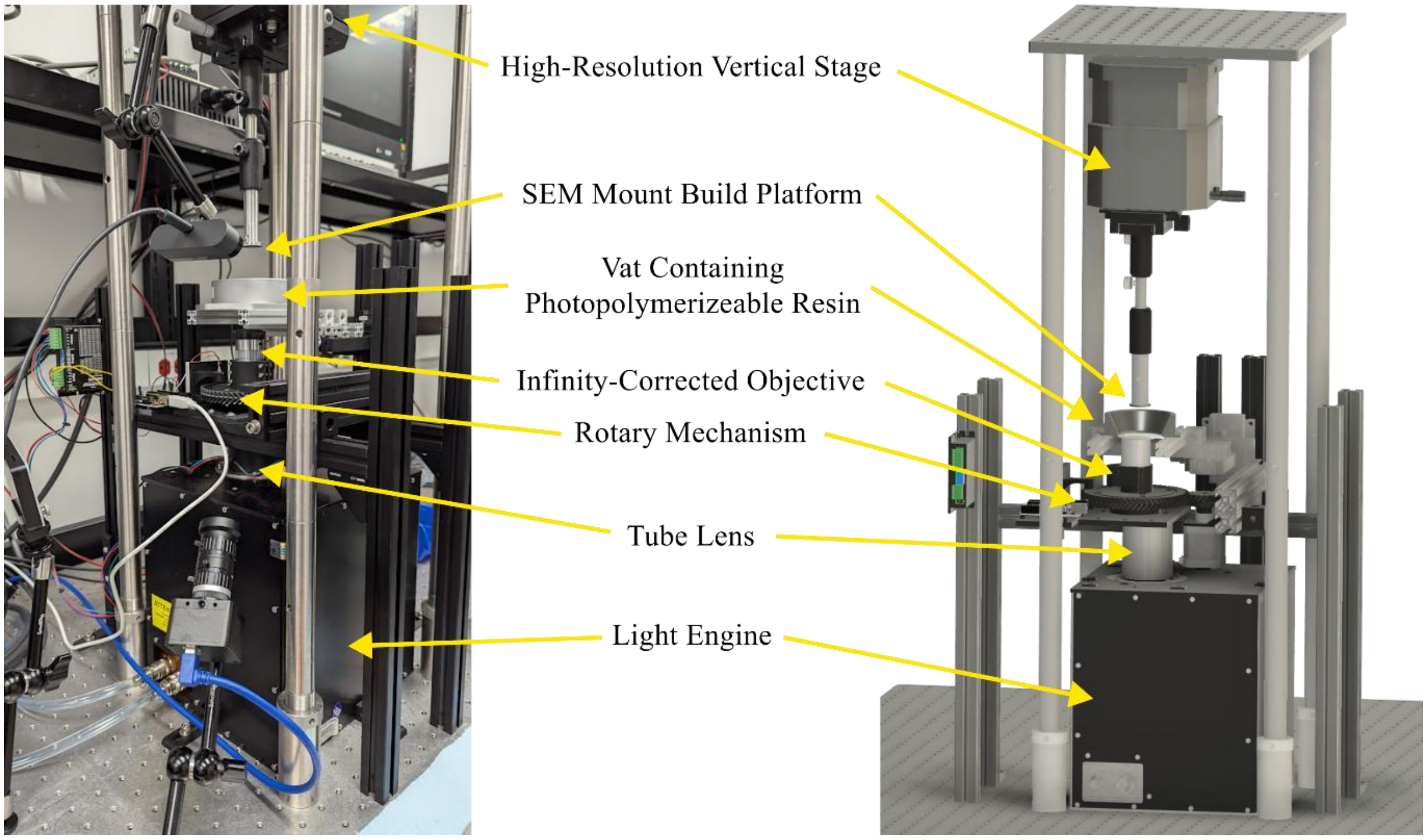

Fig.S2. Photograph of the system of the printer alongside a schematic representation.

3. Mechanical design of the system

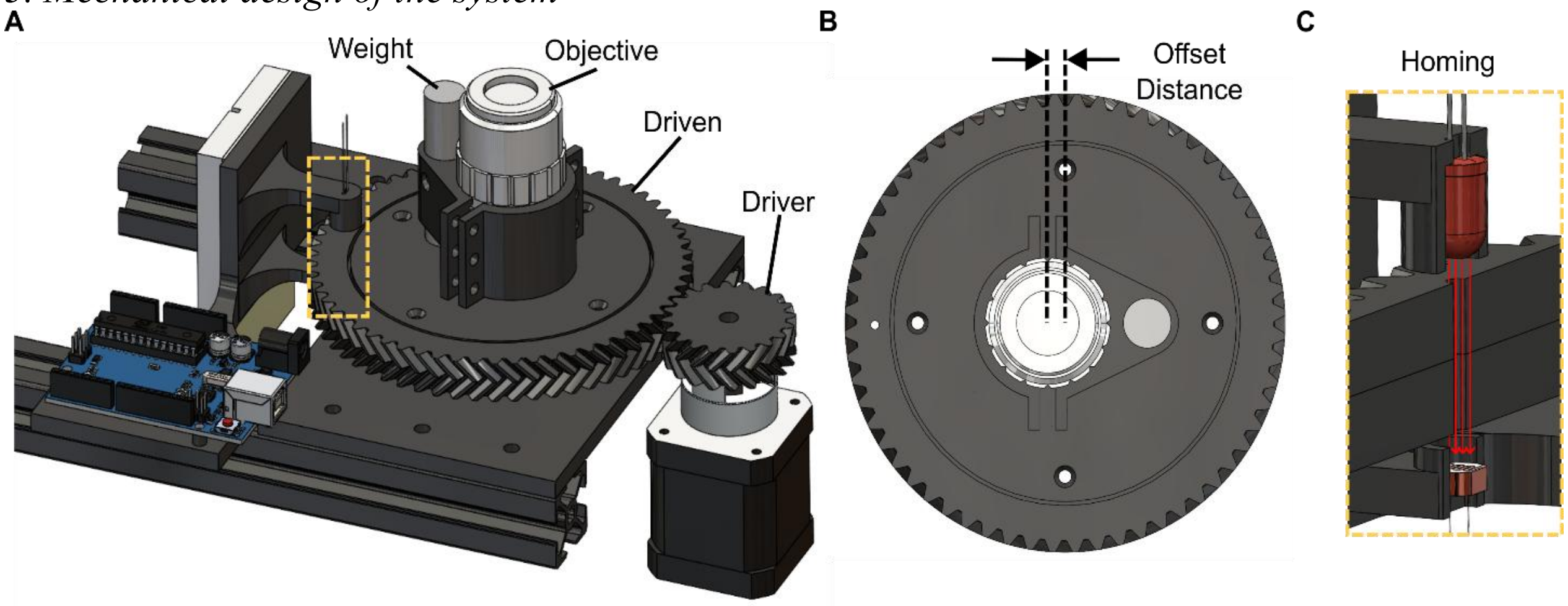

Fig. S3. Schematic representation of the SPIN system. (A) Overview of the complete system. (B) Top view of the driven gear showing the objective offset from the optical axis. (C) Illustration of the homing methodology.

To ensure the printer operates effectively, several unique features have been integrated into the system. *fig. S3A* provides an overview of this system, highlighting its two main components: the gear system and the homing system. The gear system comprises two herringbone gears, a driver and a driven gear, with a gear ratio of 1:3. The driven gear supports the objective lens and incorporates an attached counterweight to balance the objective and reduce rotational inertia. As shown in the top view (*fig. S3B*), the objective is mounted on the driven gear with a fixed offset from the optical axis, enabling the objective to orbit around the optical axis during operation.

The homing system comprises two components: a red light-emitting diode (LED) and a photoresistor, positioned on the top and bottom of the driven gear, respectively. This system establishes the 0° reference angle at the onset of projection. During the homing procedure, the LED illuminates the top surface of the driven gear, and the emitted light passes through a small aperture on the gear and is detected by the photoresistor. The resulting change in photoresistor resistance is used to identify the angular position corresponding to the 0° reference (*fig. S3C*). To improve homing accuracy, noise is minimized by averaging the photoresistor signal and confirming edge detection over consecutive readings. The rising and falling crossing angles are recorded as the aperture passes through the illumination path, with their midpoint establishing the 0° reference position. This reference position is further refined through experimental optimization, the accuracy of which is demonstrated in *fig. S11*.

4. Bill of materials

Table S1. Bill of materials for the printing system and construction of SPIN.

	Components	Details	Approx. Price
Printing system	Light Engine	9000 Firebird Light Engine, In-Vision	$20,000
	Stage	GTS70V, Newport	$9,440
	Tube lens	54-774, Edmund Optics	$950
	Objective	46-142, Edmund Optics	$1,215
		**Total price:**	**$31,605**
SPIN	3D printed parts	Driven gear, driver gear, objective holder, homing sensor mount, counterweight holder	$100
	Motor components	NEMA 17 stepper motor and shaft coupler	$25
	Control electronics	Arduino, two-phase stepper driver, breadboard, LED/photoresistor homing sensor, wires	$49
	Fasteners and hardware	Ball bearings, screws, nuts, washers, spacers, mounting hardware	$38
		**Total price:**	**$212**

5. Total build area as a function of pixel size and objective offset

The total build area of this printer setup is dependent on the pixel size at a given optical setup, p, and the objective offset from the optical axis, d. The area is a piecewise function $A(p,d)$ for three cases: 1) $d = 0$ where the build area is the native build area at a given resolution, 2) $d \leq d_{crit}$ where d_{crit} is half the image diagonal length such that the swept build area is continuous, and 3) the annular-like region case, $d > d_{crit}$, where a clove-like hole is formed in the middle of the build area (*fig. S4*).

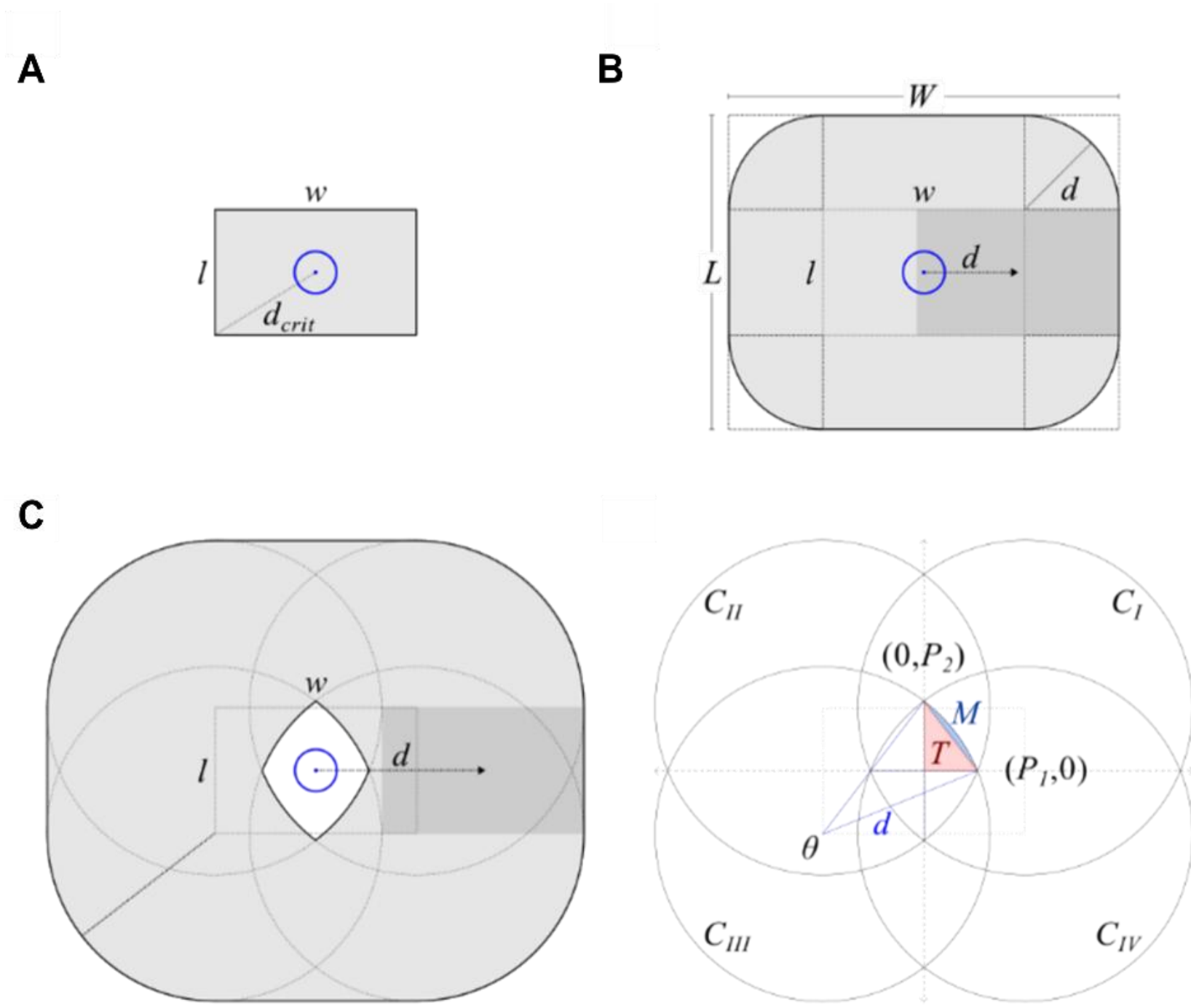

Fig. S4. Total build area as a function of pixel size and objective offset for a shifted distance equal to (A) zero, (B) half of the width, and (C) greater than half of the width.

Three cases of cumulative projected build area. In Case 1, the native build area is a rectangle defined by the optical setup, DMD, and pixel size (*fig. S4A*). In Case 2, the build area is a continuous sweep, rounded rectangle (*fig. S4B*). In Case 3, the projection is offset enough that a clove-like hole forms in the middle of the build area (*fig. S4C*). The optical axis (0,0 geometrically) is shown as a blue target.

$$A_{fab.}(p,d) = \begin{cases} A_{native} & d = 0 \\ A_{cont.} & d \leq d_{crit} \\ A_{annular} & d > d_{crit} \end{cases} \quad \textbf{(S1)}$$

$$d_{crit} = \frac{1}{2}\sqrt{w^2 + l^2} \quad \textbf{(S2)}$$

In Case 1, the build area is a rectangle of width w and length l per *Equations S3 - S4* where DMD_w and DMD_l are the DMD mirror counts in width and length, 2560 and 1600, respectively, for the light engine utilized within this work. For simplicity, let l and w be defined in terms of p:

$$w = p\mathrm{DMD}_w \quad \textbf{(S3)}$$

$$l = p\mathrm{DMD}_l \quad \textbf{(S4)}$$

This is true for the case $d = 0$ where the objective is aligned with the optical axis. The native build area is thus a rectangle of width w, length l, and area A_{native}:

$$A_{native} = lw \tag{S5}$$

In Case 2, the projection is radially offset by a distance less than half the diagonal of the projection, resulting in no hole being formed. The cumulative swept area takes the shape of a rounded rectangle via rotating the projection area around the optical axis at a constant offset. During this rotation, the projection area maintains its orientation, but its position is offset radially relative to the axis. This sweeping path is illustrated in *fig. S4B*. In this configuration, the sides of the rectangle (L, W) consist of the native projection parameters plus twice the radial offset as the rounded edges have a radius equal to the offset distance.

$$W = w + 2d \tag{S6}$$

$$L = l + 2d \tag{S7}$$

The rounded edges decrease the LW-rectangular area (A_{LW}) by a square area of side lengths $2d$ minus a circular area with radius d (A_{edges}).

$$A_{LW} = LW \tag{S8}$$

$$A_{edges} = (2d)^2 - \pi d^2 \tag{S9}$$

Thus,

$$A_{cont.} = A_{LW} - A_{edges} = lw + 2d(l + w) + \pi d^2 \tag{S10}$$

In Case 3, the projection is radially offset by a distance greater than half the diagonal of the projection area, resulting in the formation of a clove-like hole in the center. Each quadrant of the clove shape is traced by the corners of the projection area during a continuous projection sweep. This resulting shape is referred to as an annular region due to its characteristic of having a hole surrounded by a boundary, although it is non-circular in nature. The resultant area ($A_{annular}$) is the area of the no-hole case (i.e. outer edge, $A'_{cont.}$) minus the clove-like area (i.e. inner edge, A_{clove}). The outer edge can be solved as per Case 2. The clove-like area can be solved by symmetry, splitting into 4 quadrant segments such that a segment consists of a triangular region (T) plus a minor segment of a circle region (M).

$$A_{clove} = 4(T \cup M) \tag{S11}$$

Consider the clove quadrant formed in quadrant I to solve the area by symmetry. As the clove edges are formed by the vertices of the offset projection, four circles can be traced to these vertices' paths with centers at the original projection's vertices (*fig. S4C*). Thus, the triangular region in quadrant I has vertices at the optical axis (origin), P_1, and P_2. Let P be the point where the edges of two circles intersect denoted as

$$P_1 \in C_{II} \cap C_{III} \qquad\qquad P_2 \in C_{III} \cap C_{IV}$$

Consider the equations for plotting circles with centers at $C_{II}(-w/2, l/2)$, $C_{III}(-w/2, -l/2)$, and $C_{IV}(w/2, -l/2)$. P_1 and P_2 may then be found by solving each respective system of equations and taking the positive of the two solutions. A_T may then be calculated as the area of a triangle

$$A_T = \frac{1}{2} P_1 P_2 \tag{S12}$$

$$A_T = \frac{1}{2}\left(\frac{-w}{2} + \frac{1}{2}\sqrt{4d^2 - l^2}\right)\left(\frac{-l}{2} + \frac{1}{2}\sqrt{4d^2 - w^2}\right) \tag{S13}$$

The area of the minor segment (A_M) formed for circle C_{III} with radius d, bounded by points P_1 and P_2, can be calculated via the formula for a circular segment. This requires determining the central angle θ subtended by the chord $\overline{P_1 P_2}$ at the center of the circle. To compute θ, vectors are

defined from the center of C_{III} to each of the intersection points, and the dot product formula is applied to find the angle between them.

$$\theta = \cos^{-1}\left(\frac{\overrightarrow{C_{III}P_1} \cdot \overrightarrow{C_{III}P_2}}{\left|\overrightarrow{C_{III}P_1}\right|\left|\overrightarrow{C_{III}P_2}\right|}\right) = \cos^{-1}\left(\frac{\overrightarrow{C_{III}P_1} \cdot \overrightarrow{C_{III}P_2}}{d^2}\right) \tag{S14}$$

The area of the segment is then given by *Equation S15*, which accounts for the difference between the sector area and the triangular area under the chord – distinct from triangle *T*. Substituting the symbolic expressions for the vectors and simplifying yields A_M.

$$A_M = \frac{d^2}{2}(\theta - \sin\theta) \tag{S15}$$

$$A_M = \frac{d^2}{8}\left[4\cos^{-1}\left(\frac{l\sqrt{4d^2 - w^2} + w\sqrt{4d^2 - l^2}}{4d^2}\right) - \sqrt{\frac{16d^4 - \left(l\sqrt{4d^2 - w^2} + w\sqrt{4d^2 - l^2}\right)^2}{d^4}}\right] \tag{S16}$$

Thus, the area of Case 3 is

$$A_{annular} = A'_{cont.} - A_{clove} = A'_{cont.} - 4(A_M + A_T) \tag{S17}$$

The closed-form expression for the total fabrication area in terms of *w*, *l*, and *d* is thus derived. This parameterized function may be used to examine the potential build area, normalized build area, maximum fabricable circular geometry, maximum fabricable rectangular geometry, and clove circular diameter for injection-based fabrication.

The calculated build area expansion as a function of pixel size and objective offset distance is shown in *fig. S5*, with values normalized to the native build area without objective offset.

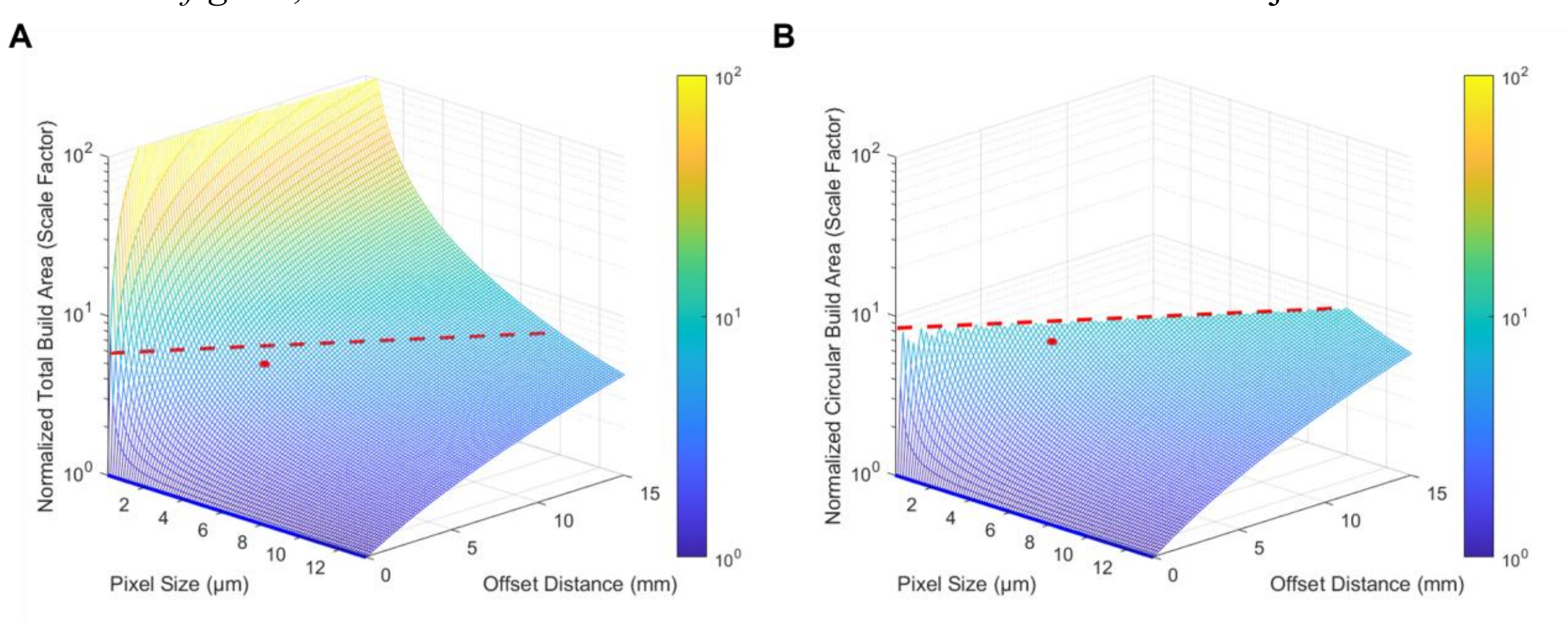

Fig. S5. Normalized build area as a function of pixel size and objective offset distance. (A) Normalized total build area. (B) Normalized circular build area. The red dashed line indicates the boundary at which the expanded build area remains continuous, without a central hole. The red dot represents the parameter set used in this work.

6. Intensity correction masks

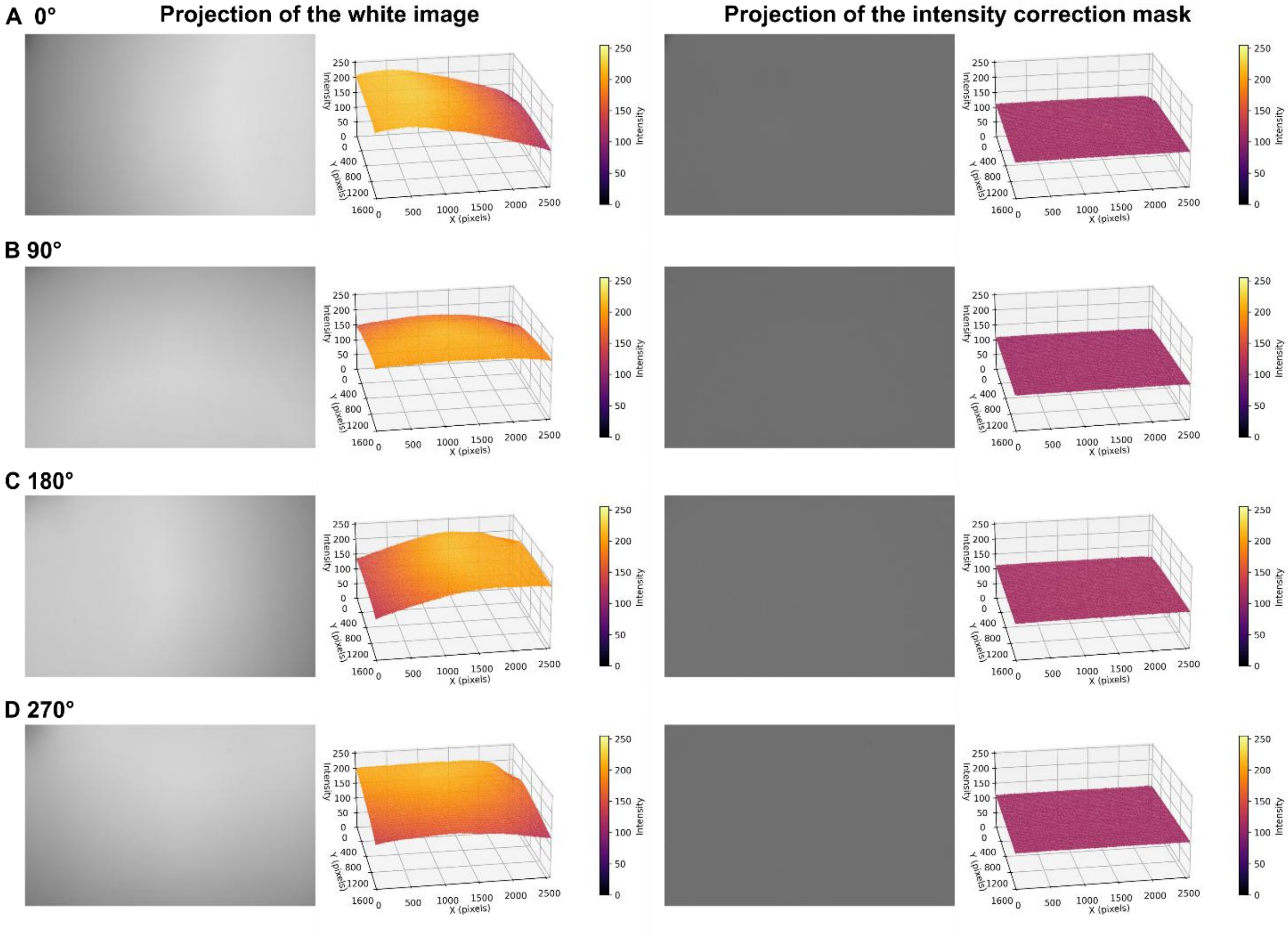

Fig. S6. Intensity correction masks. Projections of the white images and their corresponding intensity correction masks at 0° (A), 90° (B), 180° (C), and 270° (D).

7. Slanted-edge MTF analysis

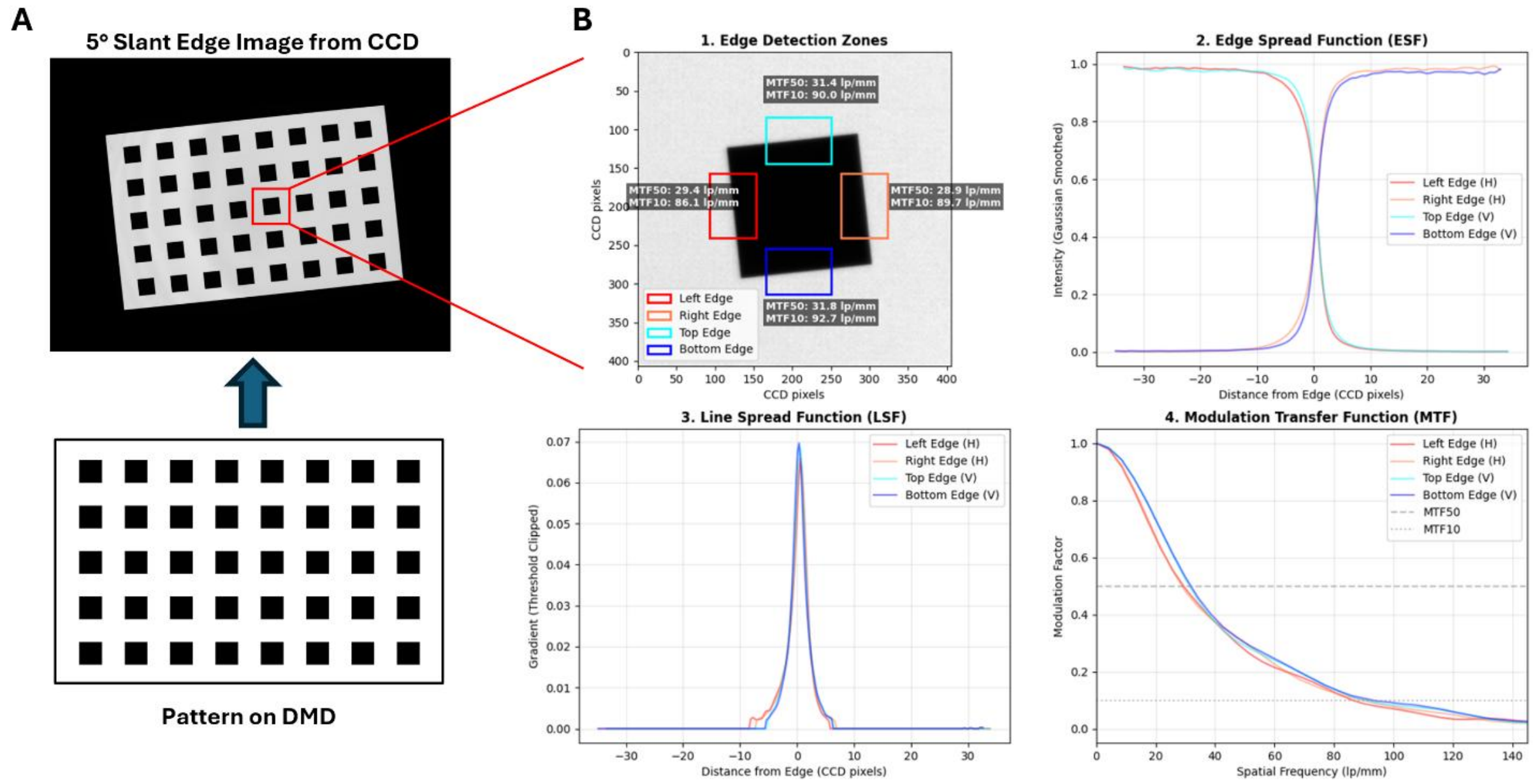

Fig. S7. Slanted-edge MTF analysis of the shift-optics system using DMD-projected targets imaged on a CCD. A perfect-edge pattern is created on the DMD at 40 distinct evenly spaced locations, which is captured by a CCD camera placed in the focal plane (A) that is rotated (slanted) by 5° about its optical axis to capture an image that is then used for slant-edge MTF analysis (B). For each CCD image, each of the 40 squares is individually cropped and processed through an automatic edge-detection algorithm (B.1), which isolates discrete regions of interest (ROIs) on the left, right, top, and bottom boundaries to assess directional performance. (B.2) For each localized ROI, pixel values across the 5° slant are projected and binned onto a one-dimensional axis to construct an oversampled, sub-pixel resolution Edge Spread Function (ESF), mapping the normalized intensity transition profile. (B.3) The first derivative of the ESF is subsequently computed to determine the Line Spread Function (LSF), defining the spatial impulse response of the optical system across the edge. (B.4) Finally, the fast Fourier transform (FFT) of the normalized LSF yields the Modulation Transfer Function (MTF) curve, plotting modulation factor against spatial frequency in line-pairs per millimeter (lp/mm). To evaluate the system's spatial frequency response across different orientations, the MTF50 and MTF10 benchmarks are extracted from the MTF profiles (B.4) at each of the 40 regions of interest for every objective rotation angle.

Table S2. Averaged MTF50 and MTF10 and half-period values for each of the rotation angles.

Objective Rotation	MTF50 (lp/mm)	MTF10 (lp/mm)	Half-period at MTF50 (µm)	Half-period at MTF10 (µm)
**0°**	29.43	84.63	17.0	5.9
**45°**	27.24	80.25	18.4	6.2
**90°**	24.69	73.49	20.3	6.8
**135°**	22.99	54.75	21.7	9.1
**180°**	23.19	64.11	21.6	7.8
**225°**	23.37	60.39	21.4	8.3
**270°**	27.68	74.87	18.1	6.7

**315°**	29.02	80.70	17.2	6.2
**Overall**	25.95	71.65	19.3	7.0

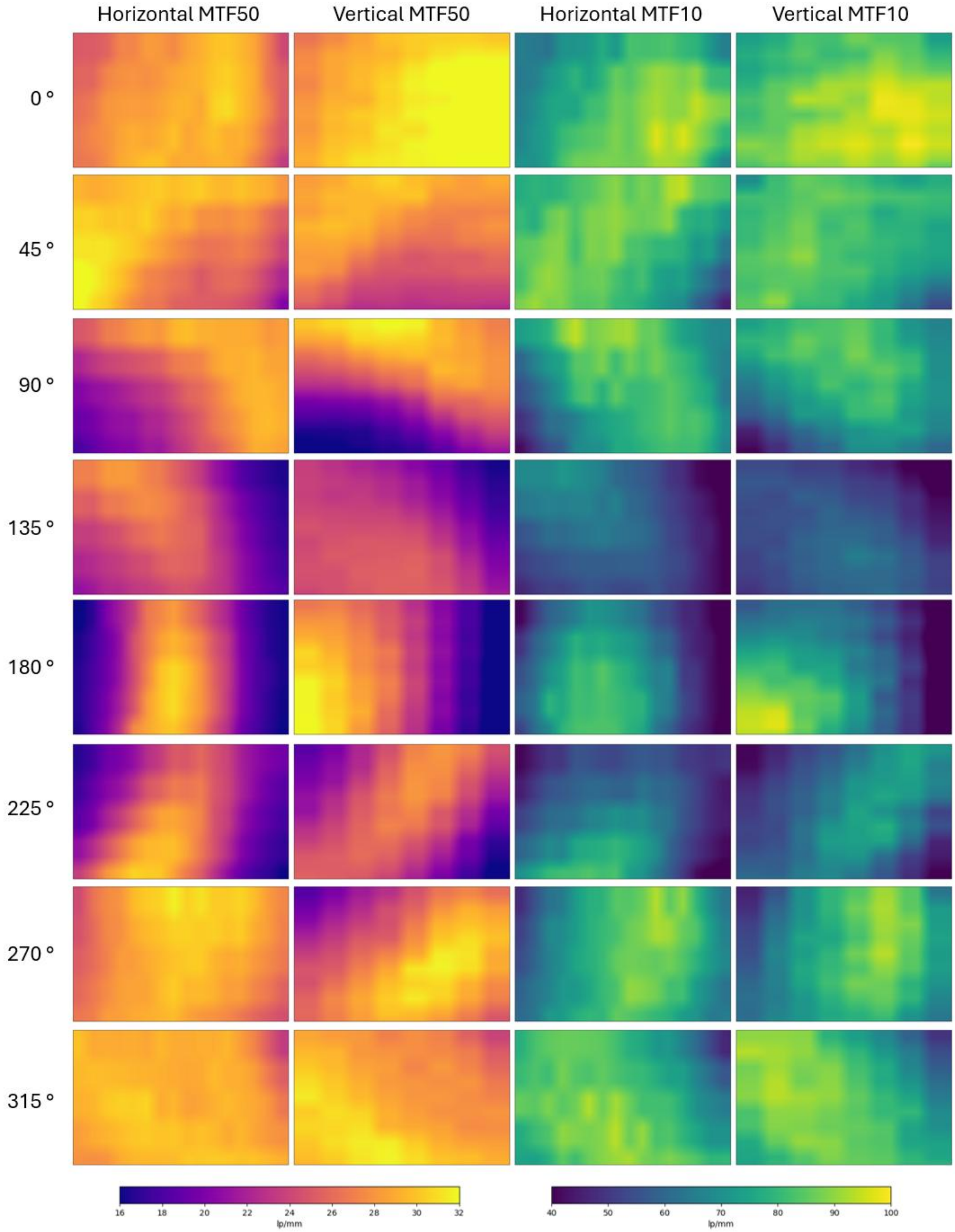

Fig. S8. Spatial field distribution of MTF50 and MTF10 performance across several target rotation angles. For each objective rotation angle, 2D spatial performance profiles are plotted as heatmaps of the horizontal and vertical components of MTF50 and MTF10, as measured via sub-pixel slant-edge technique illustrated in *fig. S7*.

8. Printing parameters for representative structures

Table S3. Printing parameters for representative structures fabricated using different SPIN printing modes.

Printed structure	Printing mode	Resin	Exposure dose (mJ/cm^2)	Layer thickness (μm)	Number of layers	Number of segments	Print time
Stanford Bunny	Single	TMPTA	6.9	16	505	505	4 min
	Basic	TMPTA	6.9	16	1163	18592	4 h 47 min
	Spiral	TMPTA	6.9	16	1163	18592	4 h 47 min
	Dynamic	TMPTA	6.9	16	1163	4500	1 h 37 min
Eiffel Tower	Single	PR48	38.7	20	686	686	12 min
	Dynamic	PR48	38.7	20	1608	4308	1 h 48 min
Octet Lattice	Single	PR48	51.6	10	297	297	15 min
	Basic	PR48	51.6	10	297	2369	1 h 18 min

9. Top view characterization of SPIN-printed lattice structures

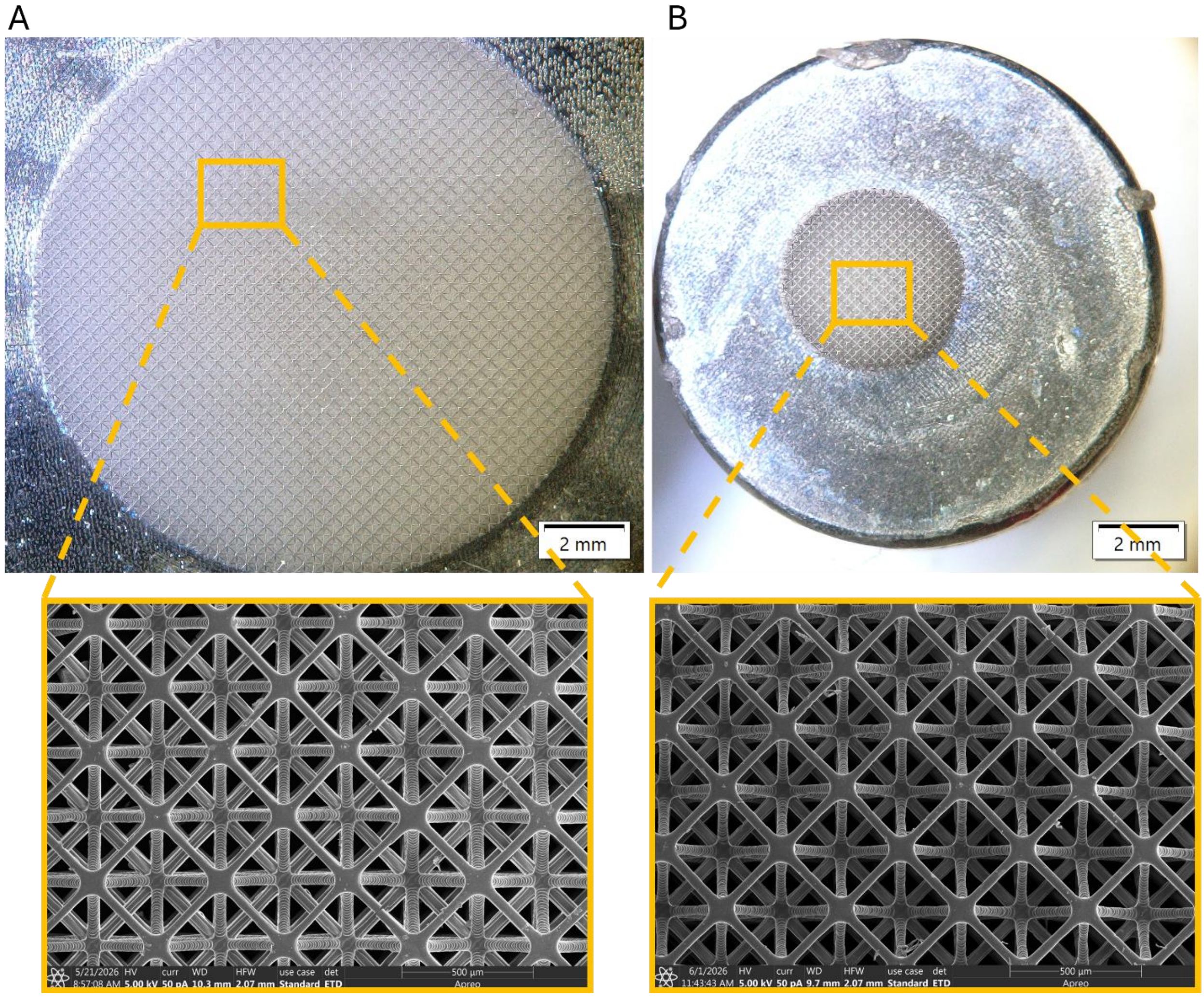

Fig. S9. Top view characterization of SPIN-printed lattice structures. (A) Images of a 15 mm diameter Octet lattice fabricated using SPIN. (B) Image of a 5 mm diameter lattice printed within the native build area. Yellow boxes indicate the regions selected for zoomed-in SEM images. SEM imaging was performed at an accelerating voltage of 5 kV and a beam current of 50 pA. Scale bars: 2 mm in the optical images and 500 µm in the SEM images.

10. Printing of Stanford bunny models with different resins

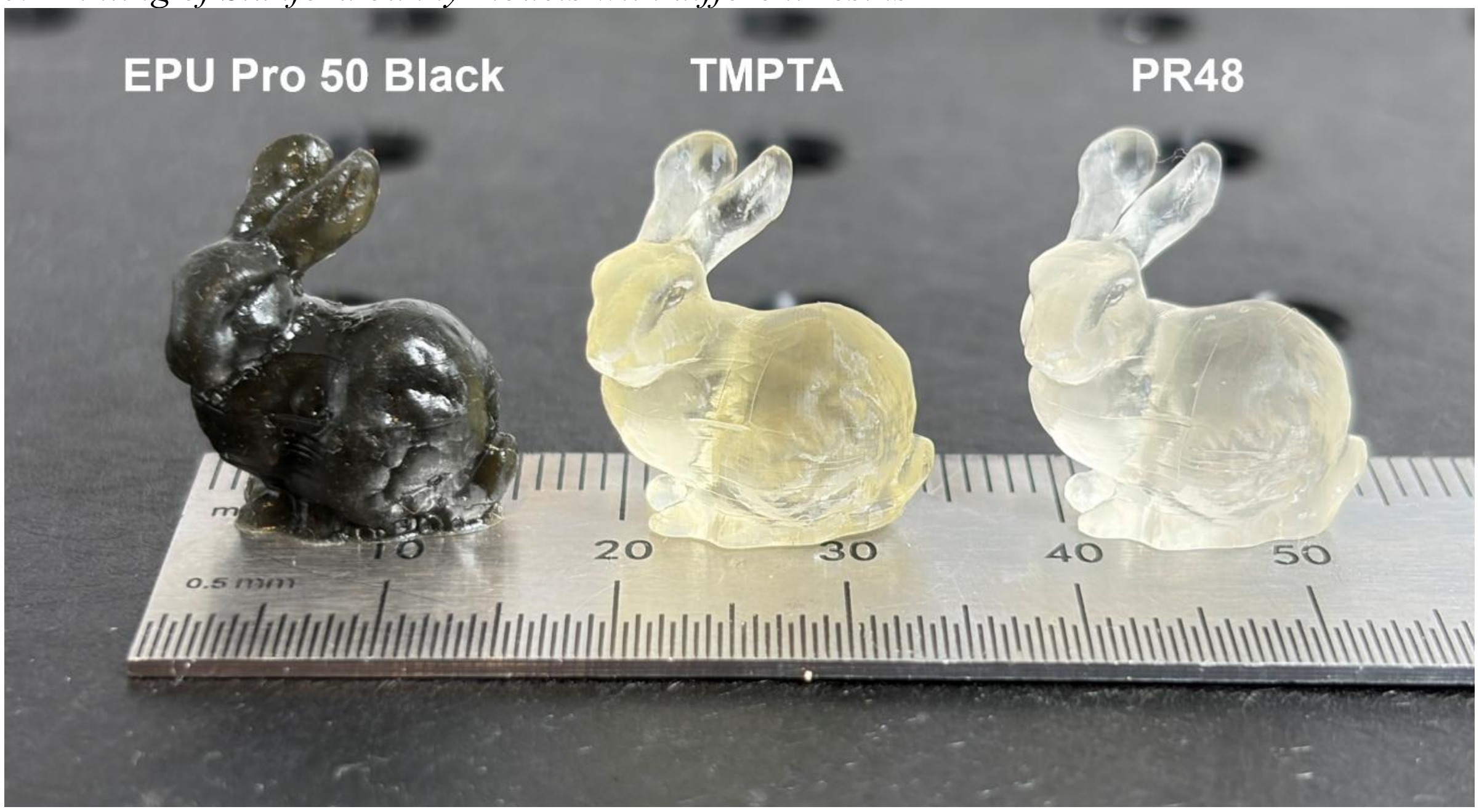

Fig. S10. Representative Stanford bunny structures printed by SPIN using EPU Pro 50 Black, TMPTA, and PR48.

Three Stanford bunny models were printed using different resins to evaluate the material compatibility of SPIN, including EPU Pro 50 Black, TMPTA, and PR48. EPU Pro 50 Black represents a particularly challenging resin for projection-based printing because of its high viscosity and demanding post-processing workflow. According to the manufacturer's datasheet(*39*), EPU Pro 50 Black has a viscosity of 1400 cP at 50 °C and requires sequential post-processing steps, including washing, moisture curing, and an inert thermal bake after printing.

TMPTA and PR48 were included as representative photopolymer resins with different curing responses. As indicated by the measured Jacob's working curves(*37*), these resins span different exposure windows and cure depth responses, demonstrating that SPIN can accommodate materials with distinct photopolymerization behavior without requiring major changes to the printer configuration.

Successful printing of the same Stanford bunny geometry across these resins highlights the robustness of SPIN for processing both standard photopolymer resins and more challenging high-viscosity elastomeric materials.

11. Verification of SPIN homing and rotation repeatability

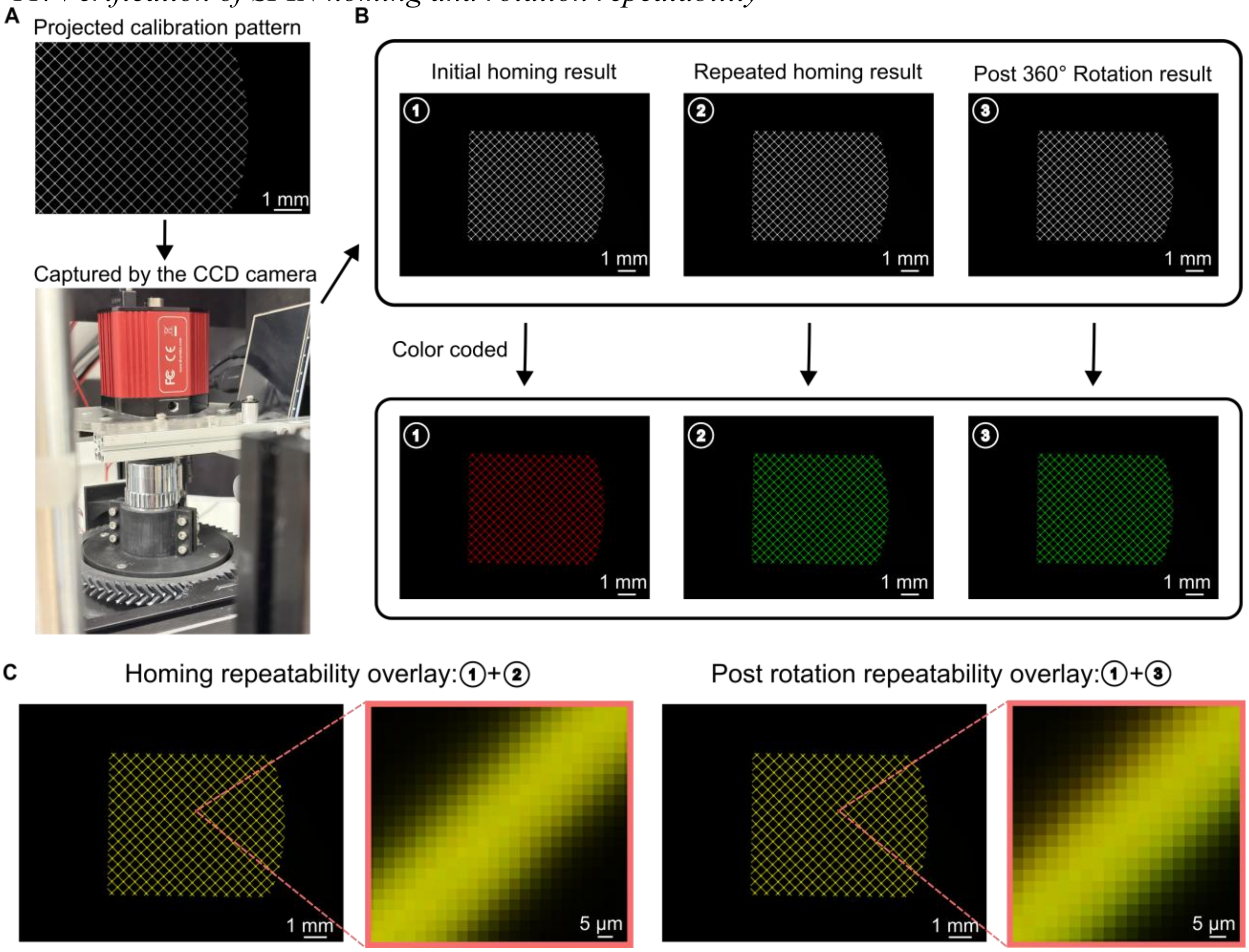

Fig. S11. Color merge verification of SPIN homing and rotation accuracy. (A) Calibration pattern projected through the SPIN optical path and captured by the CCD camera for alignment verification. (B) Representative captured images obtained at three conditions: (1) immediately after the initial homing procedure, (2) after rotating the objective to another position followed by a second homing procedure, and (3) after rotating the objective through multiple full revolutions. The initial homing image was color-coded red, while the repeated homing and post-rotation images were color-coded green for overlay comparison. (C) Overlay images used to evaluate repeatability. Strong overlap between red and green channels produces yellow features, indicating minimal positional deviation between the compared patterns. Scale bars: 1 mm in full-field images and 5 µm in zoomed-in views.

To evaluate SPIN homing and rotational repeatability, a diagonal grid pattern was projected onto a CCD camera sensor. The projected calibration pattern and the experimental setup are shown in *fig. S11A*. Three images were acquired for comparison (*fig. S11B*). The first image was captured immediately after the initial homing procedure and was used as the 0° reference position. The second image was captured after the objective was rotated to a different angular position and then homed again, providing a measurement of homing repeatability. The third image was captured after the objective was rotated through multiple full revolutions, providing a measurement of rotational positioning repeatability.

For visualization, the initial homing image was color-coded red, while the repeated homing and post-rotation images were color-coded green. When accurately aligned, the merged red and green channels appear yellow. As shown in *fig. S11C*, the overlay between the initial and repeated homing images shows strong yellow overlap, indicating high homing repeatability. Similarly, the post-rotation overlay remains well aligned with the initial homing image, confirming repeatable rotational positioning after multiple revolutions. The small red and green edge fringes indicate that the residual misalignment is within one CCD pixel, 3.45 μm. These results demonstrate high positional repeatability for both SPIN homing and rotational motion.

12. Experimental alignment optimization

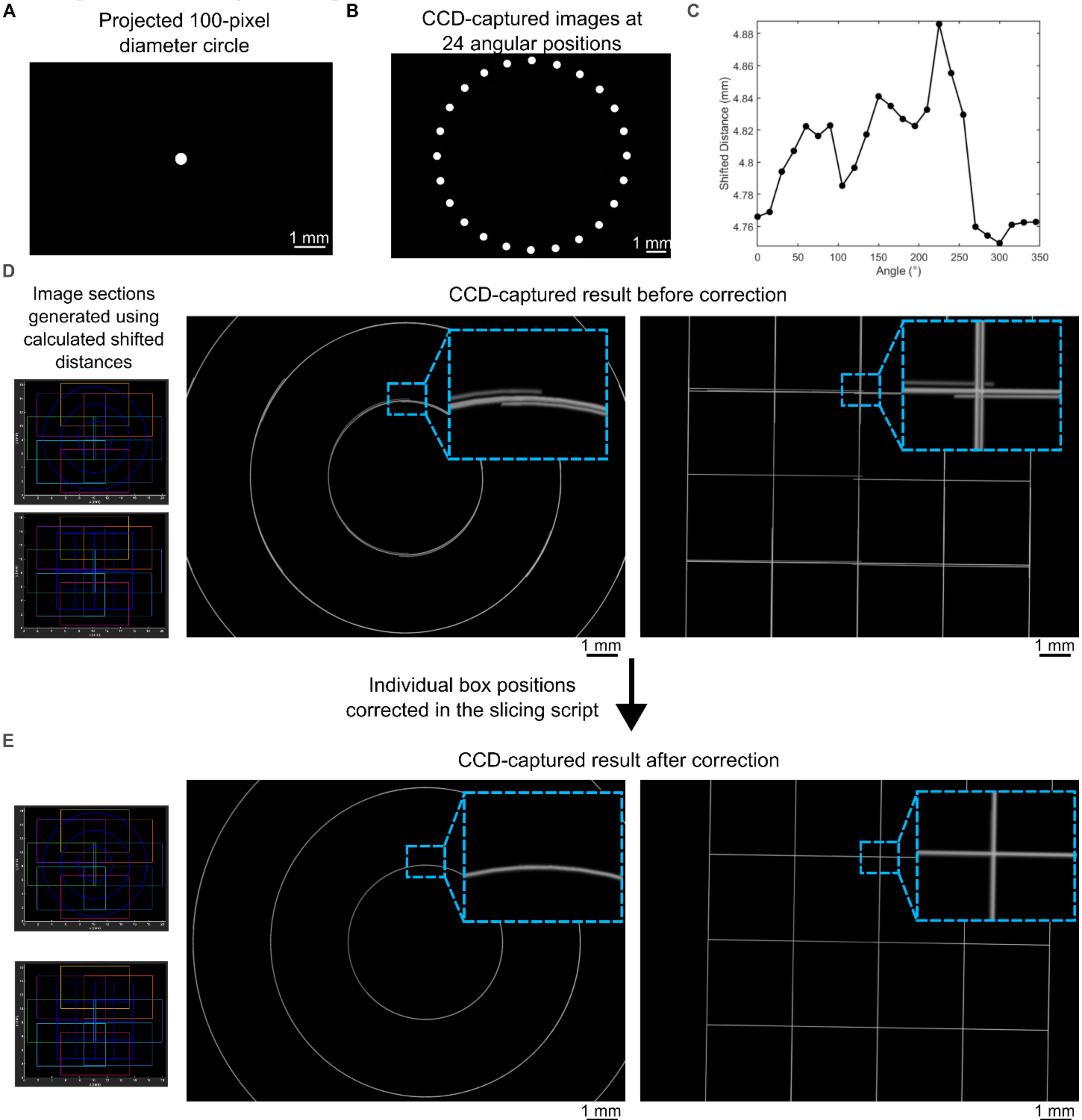

Fig. S12. Calibration of projection alignment across SPIN rotation. (A) Projected 100-pixel-diameter white circle used as the calibration marker. (B) CCD-captured positions of the projected circle at 24 angular positions. (C) Shift distance calculated from the circle fitted center as a function of rotation angle. (D) CCD-captured circle and grid calibration patterns before correction using the initially calculated shift distances. (E) CCD-captured circle and grid calibration patterns after correction using experimentally measured box positions. Scale bars: 1 mm.

To improve alignment accuracy, we performed a three-step calibration procedure. First, the image shift during SPIN rotation was experimentally measured. A 100-pixel-diameter white circle was projected at 24 angular positions and captured using a CCD camera. The center of the

24 measured circle positions was determined using MATLAB's circle-fit function, and the shift distance at each angular position was calculated relative to this fitted center.

Second, the measured shift distances were implemented in the slicing script to generate calibration patterns containing circular and rectangular grid features. These patterns were projected through SPIN and captured by the CCD camera to evaluate alignment. As shown in *fig. S12D*, the initial correction reduced the overall shift but still produced visible misalignment between projected sections.

Third, the remaining misalignment was then corrected by experimentally measuring the individual angular positions in ImageJ and updating the slicing script with these offset values. The calibration patterns were then sliced, projected, and captured using the same procedure. As shown in *fig. S12E*, the corrected circular and rectangular grid patterns show enhanced alignment across angular positions, confirming a successful alignment calibration of the SPIN system.

13. Resolution characterization of a single projection segment at 0°

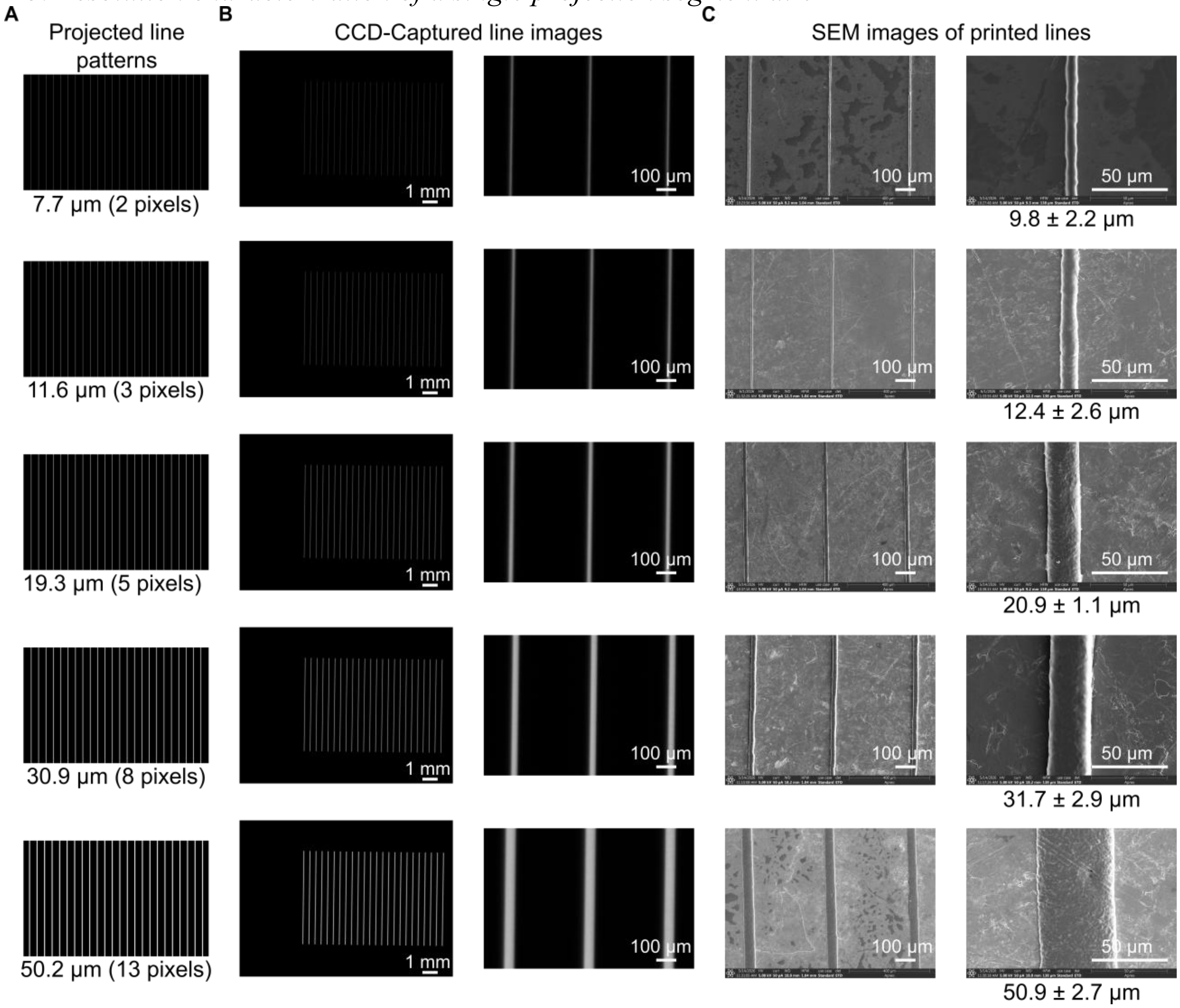

Fig. S13. Single segment resolution characterization of SPIN at 0°. (A) Projected line patterns with widths ranging from 7.7 to 50.2 μm, corresponding to 2 to 13 pixels. (B) CCD-captured images of the projected patterns and magnified views showing the resolved line features. (C) SEM images of the printed line structures and corresponding magnified views used to quantify the printed feature widths. The measured line widths were 9.8 ± 2.2, 12.4 ± 2.6, 20.9 ± 1.1, 31.7 ± 2.9, and 50.9 ± 2.7 μm for the 2-, 3-, 5-, 8-, and 13-pixel patterns, respectively. SEM imaging was performed at an accelerating voltage of 5 kV and a beam current of 50 pA. Scale bars: 1 mm for full-field CCD images, 100 μm for magnified CCD images and full-field SEM images, and 50 μm for magnified SEM images.

14. Two-pixel line patterns projected at multiple rotation angles

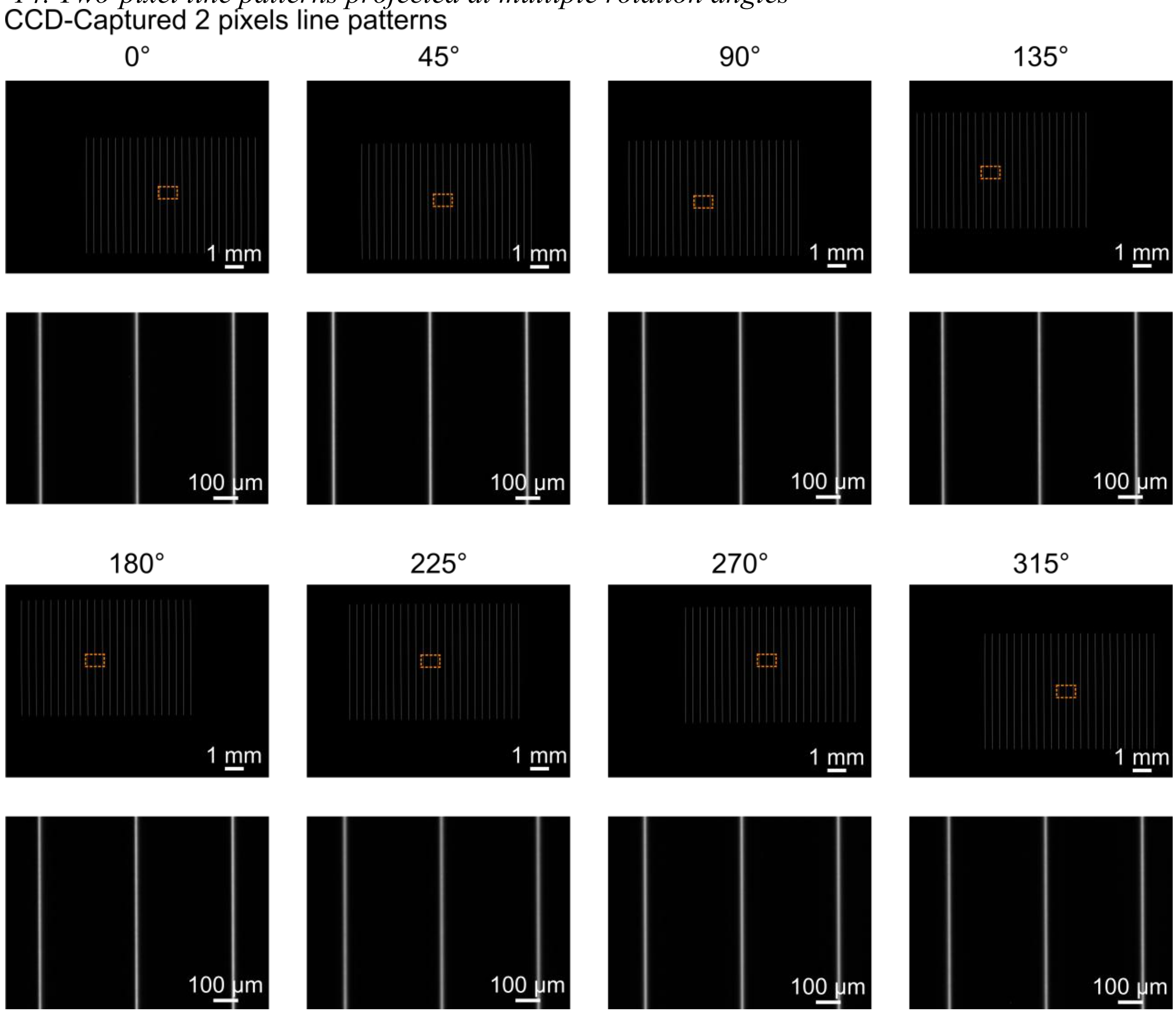

Fig. S14. Single-projection line patterns at different rotation angles. A 2-pixel-wide (7.7 µm) line pattern was projected and captured using a CCD camera at rotation angles from 0° to 315° in 45° increments. For each rotation angle, the left image shows the full CCD-captured line pattern, and the right image shows a magnified view of the region indicated by the orange dashed box. The 2-pixel line features remain distinguishable at all tested rotation angles. Scale bars: 1 mm in the full-field images and 100 µm in the magnified images.

15. Resolution characterization of overlapped projection segments

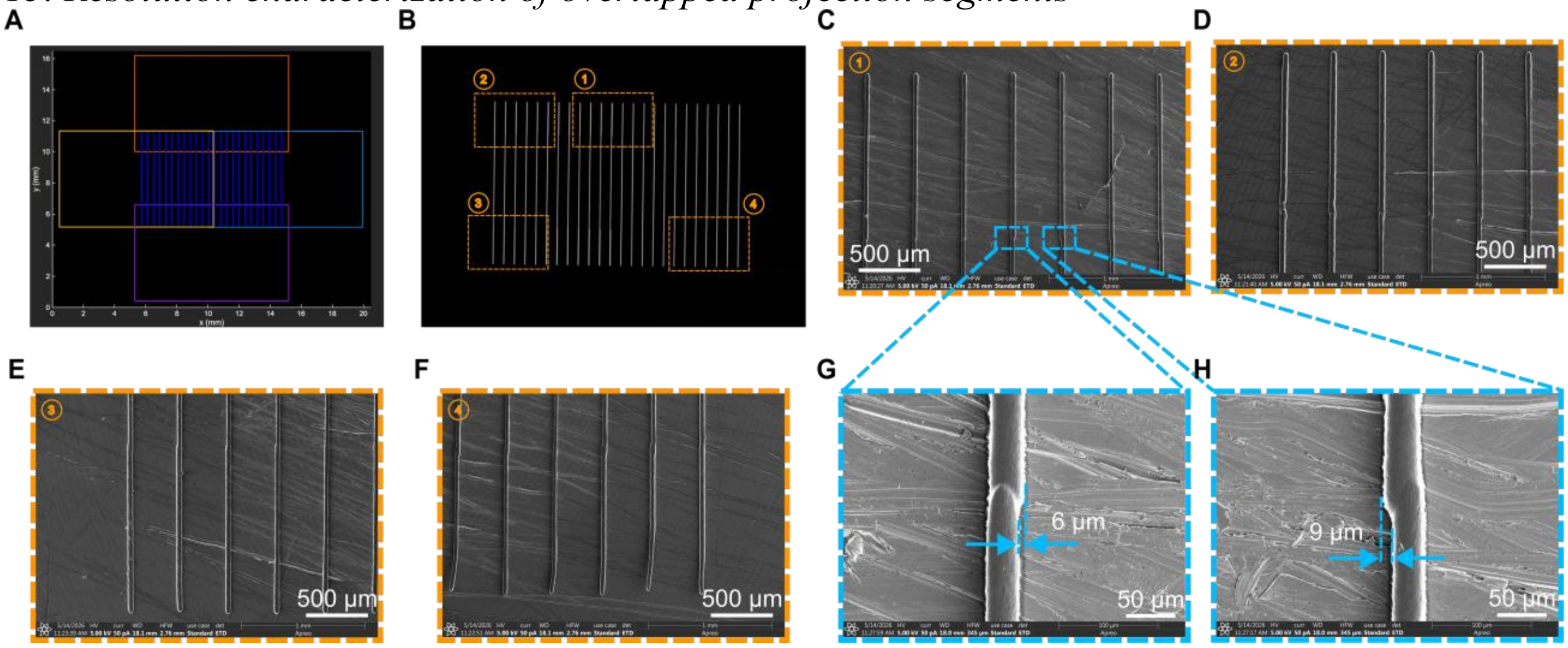

Fig. S15. Resolution and alignment characterization in overlapped projection regions. (A) An 8-pixel line pattern divided into four overlapping projection segments. (B) Merged CCD-captured image of the four segments after projection alignment. Boxed regions indicate the locations selected for SEM imaging. (C–F) SEM images showing printed line features at four representative positions across the overlapped region. (G, H) Magnified SEM views of inter-segment boundaries, where the measured misalignment is 6 μm and 9 μm, respectively. These results demonstrate sub-10 μm alignment across overlapped projection segments. SEM imaging was performed at an accelerating voltage of 5 kV and a beam current of 50 pA. Scale bars: 500 μm in overview SEM images and 50 μm in magnified SEM images.

16. Mechanical characterization of overlap regions

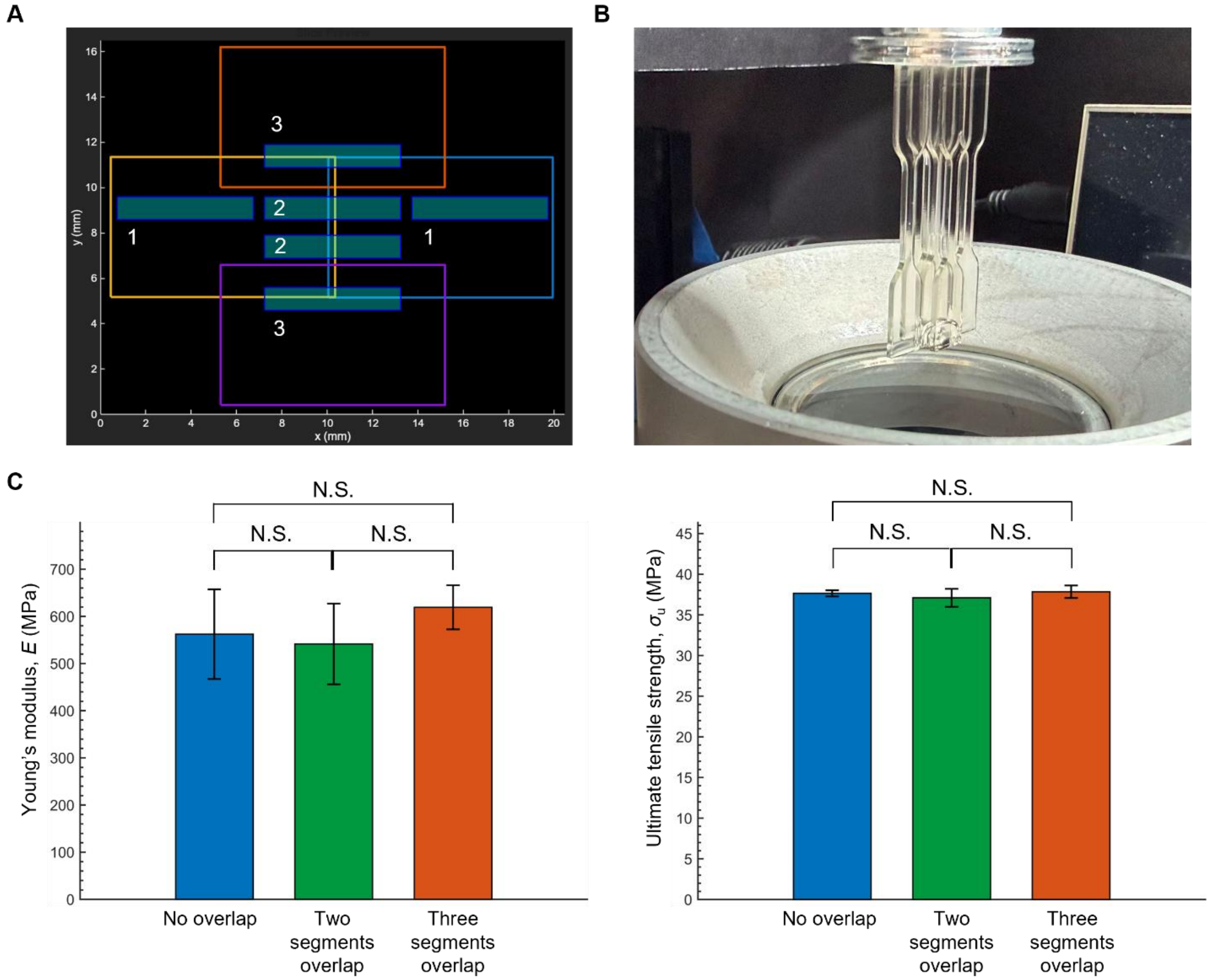

Fig. S16. Tensile testing of dog-bone specimens with different overlapping regions. (A) Sliced dog-bone design showing different overlapping regions. (B) Representative printed dog-bone specimens. (C) Young's modulus and ultimate tensile strength for the three overlap conditions. No statistically significant differences were observed among the groups, suggesting that the overlap regions maintained comparable material properties.

Supplementary Movies

Movie S1: Comparison of Printing Speed in Basic and Dynamic Segmentation Modes
Movie S2: Eiffel Tower with PR48
Movie S3: Octet Lattice with PR48

REFERENCES

1. F. Alam, N. El-Atab, Multi-material 4D printing employing stimuli-responsive polymer composites using vat photopolymerization. *Virtual Phys. Prototyp.* **20**, e2444576 (2025).

2. X. Kuang, J. Wu, K. Chen, Z. Zhao, Z. Ding, F. Hu, D. Fang, H. J. Qi, Grayscale digital light processing 3D printing for highly functionally graded materials. *Sci. Adv.* **5**, eaav5790 (2019).

3. H. Cui, D. Yao, R. Hensleigh, H. Lu, A. Calderon, Z. Xu, S. Davaria, Z. Wang, P. Mercier, P. Tarazaga, X. R. Zheng, Design and printing of proprioceptive three-dimensional architected robotic metamaterials. *Science* **376**, 1287–1293 (2022).

4. E. Rossegger, R. Höller, K. Hrbinič, M. Sangermano, T. Griesser, S. Schlögl, 3D printing of soft magnetoactive devices with thiol-click photopolymer composites. *Adv. Eng. Mater.* **25**, 2200749 (2023).

5. Z. Zhu, D. Wang, M. Zhang, L. Dong, Q. Yu, C. Jiang, X. Zhu, M. Chen, K. Zhou, G. Gu, Multimaterial 3D printed soft robots with embedded actuation and sensing. *Sci. Adv.* **11**, eadz2928 (2025).

6. X. Hu, I. C. Yasa, Z. Ren, S. R. Goudu, H. Ceylan, W. Hu, M. Sitti, Magnetic soft micromachines made of linked microactuator networks. *Sci. Adv.* **7**, eabe8436 (2021).

7. S. M. Z. S. Bukhari, M. A. Bukhari, M. Hossain, Vat photopolymerization of smart polymers for biomedical applications. *ACS Appl. Polym. Mater.* **7**, 12117–12144 (2025).

8. Z. Wang, H. Kumar, Z. Tian, X. Jin, J. F. Holzman, F. Menard, K. Kim, Visible light photoinitiation of cell-adhesive gelatin methacryloyl hydrogels for stereolithography 3D bioprinting. *ACS Appl. Mater. Interfaces* **10**, 26859–26869 (2018).

9. Y. Bao, N. Paunović, J.-C. Leroux, Challenges and opportunities in 3D printing of biodegradable medical devices by emerging photopolymerization techniques. *Adv. Funct. Mater.* **32**, 2109864 (2022).

10. N. Rajesh, J. Hwang, Y. Xu, N. Esplin, C. S. Lowenhaupt, M. Hanabusa, M. E. Szafranski, R. K. S. Shinn, C. Huang, A. V. Fesenko, S. Zhang, P. G. Schorr, R. S. S. Shinn, J. M. Perry, J. M. DeSimone, 3D-printed latticed microneedle array patches for tunable and versatile intradermal delivery. *Adv. Mater.* **36**, e2404606 (2024).

11. Y. Er, O. Güler, G. Hekimoğlu, M. Nodehi, A. Ustaoğlu, A. Sarı, O. Gencel, T. Ozbakkaloglu, Thermophysical properties and solar thermal energy storage performance of phase change composites manufactured by vat photopolymerization 3D printing technique. *J. Energy Storage* **73**, 109124 (2023).

12. X. Xu, C. Guan, L. Xu, Y. H. Tan, D. Zhang, Y. Wang, H. Zhang, D. J. Blackwood, J. Wang, M. Li, J. Ding, Three dimensionally free-formable graphene foam with designed structures for energy and environmental applications. *ACS Nano* **14**, 937–947 (2020).

13. H. Li, J. Dai, Z. Wang, H. Zheng, W. Li, M. Wang, F. Cheng, Digital light processing (DLP)-based (bio)printing strategies for tissue modeling and regeneration. *Aggregate* **4**, e270 (2023).

14. L. Wu, Y. Song, Recent innovations in interfacial strategies for DLP 3D printing process optimization. *Mater. Horiz.* **12**, 401–417 (2025).

15. J. R. Tumbleston, D. Shirvanyants, N. Ermoshkin, R. Janusziewicz, A. R. Johnson, D. Kelly, K. Chen, R. Pinschmidt, J. P. Rolland, A. Ermoshkin, E. T. Samulski, J. M. DeSimone, Continuous liquid interface production of 3D objects. *Science* **347**, 1349–1352 (2015).

16. K. Hsiao, B. J. Lee, T. Samuelsen, G. Lipkowitz, J. M. Kronenfeld, D. Ilyn, A. Shih, M. T. Dulay, L. Tate, E. S. G. Shaqfeh, J. M. DeSimone, Single-digit-micrometer-resolution continuous liquid interface production. *Sci. Adv.* **8**, eabq2846 (2022).

17. Y. Xu, H. Mao, C. Liu, Z. Du, W. Yan, Z. Yang, J. Partanen, Y. Chen, Hopping light vat photopolymerization for multiscale fabrication. *Small* **19**, e2205784 (2023).

18. M. P. Lee, G. J. T. Cooper, T. Hinkley, G. M. Gibson, M. J. Padgett, L. Cronin, Development of a 3D printer using scanning projection stereolithography. *Sci. Rep.* **5**, 9875 (2015).

19. C. Vidler, K. Crozier, D. Collins, Ultra-resolution scalable microprinting. *Microsyst. Nanoeng.* **9**, 67 (2023).

20. R. Yi, C. Wu, Y.-J. Liu, Y. He, C. C. L. Wang, Delta DLP 3-D printing of large models. *IEEE Trans. Autom. Sci. Eng.* **15**, 1193–1204 (2018).

21. Y. Wang, X. Li, S. Fan, X. Feng, K. Cao, Q. Ge, L. Gao, Y. Lu, Three-dimensional stretchable microelectronics by projection microstereolithography (PμSL). *ACS Appl. Mater. Interfaces* **13**, 8901–8908 (2021).

22. J. A. Jackson, M. C. Messner, N. A. Dudukovic, W. L. Smith, L. Bekker, B. Moran, A. M. Golobic, A. J. Pascall, E. B. Duoss, K. J. Loh, C. M. Spadaccini, Field responsive mechanical metamaterials. *Sci. Adv.* **4**, eaau6419 (2018).

23. N. A. Dudukovic, E. J. Fong, H. B. Gemeda, J. R. DeOtte, M. R. Cerón, B. D. Moran, J. T. Davis, S. E. Baker, E. B. Duoss, Cellular fluidics. *Nature* **595**, 58–65 (2021).

24. X. Zheng, W. Smith, J. Jackson, B. Moran, H. Cui, D. Chen, J. Ye, N. Fang, N. Rodriguez, T. Weisgraber, C. M. Spadaccini, Multiscale metallic metamaterials. *Nat. Mater.* **15**, 1100–1106 (2016).

25. M. Kim, G. Yoo, B. Kim, Y. Song, B. J. Lee, Scalability enhancement in projection-based 3D printing through optical expansion. *Addit. Manuf.* **95**, 104511 (2024).

26. The MathWorks Inc., Optimization Toolbox User’s Guide, R2025b (2025); https://mathworks.com/help/optim/.

27. P. F. Jacobs, *Rapid Prototyping & Manufacturing: Fundamentals of Stereolithography* (Society of Manufacturing Engineers, 1992).

28. J. Bennett, Measuring UV curing parameters of commercial photopolymers used in additive manufacturing. *Addit. Manuf.* **18**, 203–212 (2017).

29. Z. D. Pritchard, M. P. de Beer, R. J. Whelan, T. F. Scott, M. A. Burns, Modeling and correcting cure-through in continuous stereolithographic 3D printing. *Adv. Mater. Technol.* **4**, 1900700 (2019).

30. P. R. Onffroy, S. Chiovoloni, H. L. Kuo, M. A. Saccone, J. Q. Lu, J. M. DeSimone, Opportunities at the intersection of 3D printed polymers and pyrolysis for the microfabrication of carbon-based energy materials. *JACS Au* **4**, 3706–3726 (2024).

31. Z. J. Geffert, Z. Xiong, J. Grutzmacher, M. Wilderman, A. Mohammadi, A. Filip, Z. Li, P. Soman, Multipath projection stereolithography for three-dimensional printing microfluidic devices. *ACS Appl. Mater. Interfaces* **16**, 69807–69817 (2024).

32. K. C. H. Chin, G. Ovsepyan, A. J. Boydston, Multi-color dual wavelength vat photopolymerization 3D printing via spatially controlled acidity. *Nat. Commun.* **15**, 3867 (2024).

33. I. A. Coates, W. Pan, M. A. Saccone, G. Lipkowitz, D. Ilyin, M. M. Driskill, M. T. Dulay, C. W. Frank, E. S. G. Shaqfeh, J. M. DeSimone, High-resolution stereolithography: Negative spaces enabled by control of fluid mechanics. *Proc. Natl. Acad. Sci. U.S.A.* **121**, e2405382121 (2024).

34. G. Lipkowitz, T. Samuelsen, K. Hsiao, B. Lee, M. T. Dulay, I. Coates, H. Lin, W. Pan, G. Toth, L. Tate, E. S. G. Shaqfeh, J. M. DeSimone, Injection continuous liquid interface production of 3D objects. *Sci. Adv.* **8**, eabq3917 (2022).

35. M. S. Fischer, Cost-effective, open-source light shutters with Arduino control. *HardwareX* **19**, e00548 (2024).

36. The MathWorks Inc., Image Processing Toolbox User’s Guide, R2025b (2025); https://mathworks.com/help/images/index.html.

37. J. M. Kronenfeld, L. Rother, M. A. Saccone, M. T. Dulay, J. M. DeSimone, Roll-to-roll, high-resolution 3D printing of shape-specific particles. *Nature* **627**, 306–312 (2024).

38. J. Schindelin, I. Arganda-Carreras, E. Frise, V. Kaynig, M. Longair, T. Pietzsch, S. Preibisch, C. Rueden, S. Saalfeld, B. Schmid, J.-Y. Tinevez, D. J. White, V. Hartenstein, K. Eliceiri, P. Tomancak, A. Cardona, Fiji: An open-source platform for biological-image analysis. *Nat. Methods* **9**, 676–682 (2012).

39. Carbon Inc., “EPU Pro 50 and 90 Black Technical Datasheet” (Carbon Inc., 2026).
